# Explainable Ensemble Learning for Robust Severity Stratification of Carpal Tunnel Syndrome from Clinical Data

**DOI:** 10.3390/diagnostics16111604

**Published:** 2026-05-25

**Authors:** Muhammet Emin Sahin, Hasan Ulutas, Murat Korkmaz, Mucella Ozbay Karakus, Orhan Er, Huriye Unluel

**Affiliations:** 1 Department of Computer Engineering, Izmir Bakircay University, 35665 Izmir, Turkey; 2 Department of Computer Engineering, Yozgat Bozok University, 66100 Yozgat, Turkey; 3 Department of Orthopedics and Traumatology, Faculty of Medicine, Yozgat Bozok University, 66100 Yozgat, Turkey; 4 Department of Neurology, Faculty of Medicine, Yozgat Bozok University, 66100 Yozgat, Turkey

**Keywords:** carpal tunnel syndrome (CTS), machine learning, data augmentation, stacking ensemble, SHAP, UN SDG 3

## Abstract

**Background/Objectives:** This paper aims to design an explainable and accurate ML framework to support the automatic classification of Carpal Tunnel Syndrome (CTS) severity from structured patient data. **Methods:** For the experiment, an open-source dataset of 1037 samples was used. Following stratified partitioning, 305 samples were held out as the test set; the remaining training set (*n* = 732) was augmented to 1216 balanced samples via ADASYN, yielding an 80/20 train/test ratio relative to the final dataset (*n* = 1521). In order to solve the problem of imbalance associated with CTS cases of moderate and severe severity, the Adaptive Synthetic Sampling (ADASYN) technique was employed. The model’s predictive capacity was increased by means of feature engineering methods, such as polynomial transformations and clinically relevant interactions. Specifically, four ensemble learning models (XGBoost, Random Forest, LightGBM, and CatBoost) were optimized and ensembled with the use of a stacking approach with a base algorithm of LightGBM. The explainability of the model was ensured through SHAP and LIME analysis. **Results:** As a result, the stacking ensemble was able to reach a test accuracy of 91.15%, an F1-score of 91.13%, and an ROC-AUC of 0.9708. The proposed ensemble performed superiorly compared to any other individual algorithm while having stable performance across all severity categories. **Conclusions:** Through the explainability analysis, it was observed that such a classification model relies on important clinically relevant predictors, including cross-sectional area (CSA), duration of symptoms, pain level measured by the numeric rating scale of pain (NRS), and palmar bowing (PB).

## 1. Introduction

Carpal Tunnel Syndrome (CTS) constitutes a relatively frequent peripheral neuropathy characterized by the compression of the median nerve at the wrist. Accurate diagnosis and correct classification of severity are vital for adequate management and subsequent treatment plans. Though clinical exam and neurophysiological measurements (e.g., NCS and EMG) represent the gold standard for assessing the severity of CTS, these methods suffer from certain drawbacks related to high inter-rater variability, a time-consuming nature, and low reproducibility. Recently, there has been a surge of interest in employing ML and DL-based models for the diagnosis and severity classification in CTS. Among conventional severity classification schemes, the Padua electrophysiological classification scale and the Boston Carpal Tunnel Questionnaire are currently the two most commonly used ones [1,2]. Nonetheless, due to the primary reliance on neurophysiological findings, the latter could lead to certain discrepancies with clinical symptoms, resulting in decreased diagnostic accuracy. This problem encouraged researchers to focus on a more objective classification based on multi-source data fusion. The combined use of electrophysiological tests and diagnostic ultrasonography is increasingly gaining popularity as a method of objective assessment of carpal tunnel syndrome (CTS) severity. According to systematic reviews, the median nerve cross-sectional area (CSA) measurement provides a level of diagnostic accuracy comparable to the electrodiagnostic test with additional advantages such as pain-free examination, the possibility of visualization, and availability in outpatient clinics [3]. Besides morphometric characteristics, other quantitative ultrasonic parameters, such as nerve echogenicity, intraneural vascularity measured with the Doppler technique, and a ratio between wrist and forearm CSAs, have shown independent correlation with the severity grade assessed with electrophysiology [4,5]. Despite the advances in the diagnosis of CTS, there remains a problem of significant inter-observer and inter-center variation when using ultrasound in assessing the severity grade, thus limiting the ability to obtain standardized results in diverse settings [3]. The unmet need for standardization and interpretability of ultrasound and clinical parameters has formed the basis for the development of machine learning technology discussed in the current work. Several recent studies revealed that ML algorithms, such as Random Forest, SVM, and XGBoost, could classify the severity of CTS on the basis of combined neurophysiological, clinical, and anthropometric characteristics [6,7,8,9,10]. Elseddik et al. trained ML models on multiple neurophysiological parameters and achieved over 90% accuracy in differentiating the severity levels [7]. Inui et al. utilized a gradient boosting approach to predict post-operative neurophysiological improvement and CTS Instrument (CTSI) score change [8]. In addition, Bakalis et al. compared the results of motor and sensory NCS and optimized classification performance by applying sensor-based metrics [11]. Another powerful way of modeling complex data was offered by DL models. Beyond structured data, deep learning techniques have also been applied to medical image fusion tasks—such as discrete wavelet transform-based approaches—demonstrating strong feature extraction capabilities across multimodal clinical imagery [12]. Elseddik et al. employed CNN and LSTM models to classify severity on the basis of neurophysiological parameters, showing enhanced sensitivity in detecting severe cases of CTS [13]. Multimodal methods have also attracted significant attention in the last couple of years. For instance, Yetis et al. utilized anthropometric features and grip strength parameters in the classification of CTS severity using multiple ML algorithms [14]. Sasaki et al. conducted a large-scale validation of the Padua and Bland classifications on over 1100 hands. They showed a statistically significant negative correlation between sensory conduction velocity and distal motor latency; additionally, the researchers pointed out some classification gaps in both scales, which indicated a relative subjectivity in the current standards [15]. Pellicer-Valero et al. applied spectral clustering for revealing heterogeneous profiles of central sensitization in patients with CTS [16]. Wei et al. proposed hybrid ML models incorporating clinical symptom scores and neurophysiological data [17]. Moreover, Lyu et al. analyzed the application of ultrasound-derived radiomic features for severity prediction. The authors used a Random Forest model and were able to achieve greater classification accuracy than the conventional method of median nerve cross-sectional area estimation (CSA). Particularly, the model could classify mild, moderate, and severe CTS with 76.39% accuracy in the test set [18]. Oten et al. employed a six-dimensional EMG-derived feature space with classifiers including Decision Tree, SVM, and ANN, demonstrating that ANOVA-based feature selection consistently improved classification performance across all methods, with the Decision Tree achieving 99.49% accuracy in a reduced feature space [19]. In conclusion, the current research literature suggests that there is parallel development of electrophysiological assessment methods and ML algorithms in CTS severity classification. Combining different sources of data, such as clinical, functional, and imaging data, promises to yield more objective, accurate, and clinically meaningful methods for evaluating the severity of the condition. Based on the existing literature gaps and breakthroughs mentioned above, this study attempts to develop an ML-based system for the classification of CTS severity, incorporating neurophysiological, clinical, and hand function data. Particular emphasis will be placed on evaluating performance for different severity stages. The main contributions of the current study are as follows:

- The findings were compared to baseline values obtained by Park et al. [6] in the original work introducing the dataset to verify the efficiency of our data augmentation scheme. Specifically, with no oversampling and using only the classical ML algorithm (XGBoost), the highest accuracy in their analysis amounted only to 76.6%. In contrast, our data augmentation approach, namely ADASYN, significantly boosted the performance to 90.45%, yielding an approximate 14% improvement over baseline values.
- In contrast to existing ensemble structures based on stacking, an innovative stacking ensemble framework using XGBoost, Random Forest, LightGBM, and CatBoost, combined with calibrated probability aggregation, was developed and optimized. This model obtained 91.15% accuracy while keeping an optimal balance in performance for all three CTS severity levels (Mild/Moderate/Severe). Thus, this method directly addresses the need for multi-class stratification in the diagnosis of CTS severity, which has been overlooked by previous studies concentrating primarily on binary classification.
- The use of feature engineering, such as polynomial transformations, interaction terms, and categorical binning, which is clinically motivated, is incorporated in the stacking ensemble structure. It improves the ability of the model to distinguish between different severity levels and makes the results more interpretable for clinicians.
- To solve the problem of a lack of quantitative consistency evaluation between global and local explainable AI methods, both SHAP and LIMEs are utilized and compared. Consistent results from both models prove that clinically relevant features, such as cross-sectional area (CSA), symptom duration, and pain intensity, are the most influential factors for the decision-making process of the model.
- Besides accuracy metrics, we also analyzed macro-average metrics, ROC/PR AUC, the Matthews Correlation Coefficient (MCC), and Cohen’s Kappa. Visualization of dimensionality reduction (PCA and t-SNE) and feature importance helped in confirming the model’s stability.

The remainder of this paper is organized as follows. Section 1 reviews related work on ML-based CTS diagnosis and severity classification, providing a comparative analysis with existing approaches. Section 2 describes the materials and methods, including the dataset, data augmentation strategies, feature engineering procedures, ML algorithms, stacking ensemble architecture, and explainability framework. Section 3 presents the experimental results, including ablation analysis, statistical comparisons, and XAI findings. Section 4 provides a comprehensive discussion of results in the context of clinical relevance and study limitations. Section 5 concludes with a summary of contributions and future research directions.

### Related Works

The performance of other machine learning and deep learning models for diagnosing CTS and determining severity is presented in Table 1. Previous research has mostly concentrated on binary classification problems or image-based detection with a restricted number of samples. In comparison, the current study improves upon previous models by employing a larger total dataset (*n* = 1521; training set augmented to 1216 via ADASYN, test set *n* = 305), sophisticated ensemble modeling using stacking, and incorporating explainability methods (SHAP and LIME). With a final accuracy score of 91.15%, the suggested approach surpasses many existing models regarding classification effectiveness, particularly in multiclass severity prediction—a far more difficult problem compared to binary classification.

## 2. Materials and Methods

### 2.1. Dataset

In this research, we employed a publicly available, well-annotated dataset, which was created for classification purposes through machine learning algorithms regarding the severity of CTS. This dataset was built upon the retrospective analysis carried out using various parameters extracted from the medical records of 1037 patients with CTS-affected hands. In total, there were 11 variables used for each record in the dataset, including both demographic parameters (e.g., age, gender, and body mass index), clinical (e.g., the length of symptoms, NRS score, presence of night pains, and presence of thenar muscle atrophy) and sonographic parameters (e.g., CSA of median nerve and PB of the flexor retinaculum) [6]. The lack of publicly available, well-organized datasets on CTS still remains one of the critical barriers for developing advanced machine learning-based diagnostic systems. Beyond access limitations, clinical datasets frequently suffer from class imbalance, particularly the underrepresentation of moderate and severe CTS cases. We address this problem by employing several methods of data augmentation aimed at solving the class imbalance problem. Further on, after this stage of preprocessing, ML models were developed to enhance their classification capacity for CTS severity prediction. The proposed technique provides an opportunity to create more balanced working conditions for ML models and increase the reliability of their results. It should be mentioned that the focus was on the underrepresented categories. A brief overview of baseline demographic and clinical features is provided in Table 2.

### 2.2. Data Augmentation Methods

Data augmentation refers to various methodologies used to increase the diversity of a dataset by producing more data instances based on the existing ones and retaining their key properties. Even though it gained widespread popularity in the field of computer vision, data augmentation has shown its effectiveness in the area of structured medical datasets, especially when it comes to handling class imbalance problems and increasing the generalization ability of machine learning models [29,30]. Data augmentation techniques, including synthetic data creation and oversampling of rare classes (e.g., SMOTE and ADASYN), have been widely used in the context of healthcare due to restrictions in acquiring additional medical data due to costs and ethical considerations [31,32]. For this study, ADASYN was selected to deal with an intrinsic class imbalance in the severity of the CTS dataset. ADASYN allowed for the creation of synthetic examples for rare severity levels, which made it possible to balance the distribution of mild, moderate, and severe cases. The use of ADASYN was indispensable in improving the predictive power of the classifier to work consistently in any severity class, especially in rare ones.

### 2.3. Machine Learning (ML)

ML techniques are essentially mathematical techniques that involve pattern recognition and prediction using learning methods from the structured database in order to guide decision-making processes [33,34,35]. In particular, when used in health care applications, such ML techniques have proven useful in handling high-dimensional data in order to identify non-linear relationships. In this study, several high-performance ML algorithms were employed, including XGBoost, Random Forest, LightGBM, and CatBoost as base learners, along with a LightGBM-based stacking meta-learner. These algorithms were applied to processed and balanced clinical datasets to construct predictive models, optimize decision boundaries, and enhance classification performance. Within the scope of diagnostic support for CTS severity classification, the use of these ensemble and boosting-based methods improved predictive accuracy, ensured balanced performance across all classes, and increased the reliability of the proposed clinical decision support framework.

### 2.4. Evaluation Metrics

The performance of the proposed classification framework was assessed using an extensive set of evaluation measures that would allow for a thorough examination of the model’s behavior for each of the three categories of CTS severity. Since the problem under consideration is multiclass and faces a challenge of class imbalance common in clinical data, assessing the performance of the model using a single metric would not be sufficient [33,34,36,37]. Therefore, a suite of complementary metrics was employed, each capturing a distinct aspect of classification performance. The metrics are detailed below:

(1) $$\mathrm{Accuracy}=\frac{TP+TN}{TP+TN+FP+FN}$$

(2) $$\mathrm{Precision}=\frac{TP}{TP+FP}$$

(3) $$\mathrm{Recall}=\frac{TP}{TP+FN}$$

(4) $$F_{1}=2\times \frac{\mathrm{Precision}\times \mathrm{Recall}}{\mathrm{Precision}+\mathrm{Recall}}$$

(5) $$MCC=\frac{TP\times TN-FP\times FN}{\sqrt{\left(TP+FP)(TP+FN)(TN+FP)(TN+FN\right)}}$$

(6) $$\kappa =\frac{p_{o}-p_{e}}{1-p_{e}}$$

SHAP [38] assigns each feature i a Shapley value $\phi_{i}$ that quantifies its marginal contribution to the model output, computed over all possible feature subsets S⊆F∖{i}:

(7) $$\phi_{i}\left(f\right)=\sum_{S\subseteq F\setminus \left\{i\right\}}\frac{|S|!(|F|-|S|-1)!}{|F|!}\left[f\left(S\cup \left\{i\right\}\right)-f\left(S\right)\right]$$

where F is the full feature set, and f(S) is the model output using only the features in subset S. This formulation guarantees properties of local accuracy, missingness, and consistency, making SHAP values theoretically grounded for global feature importance analysis. LIMEs [39] approximate the black-box model f locally around a given instance x by fitting an interpretable surrogate model g from an explanation family G. The optimal surrogate is obtained by solving:

(8) $$g^{*}=\arg\;\min\;g\in G\;L\left(f,g,\pi_{x}\right)+\Omega \left(g\right)$$

where L measures the fidelity of g to f in the neighborhood of x, $\pi_{x}$ is a proximity kernel that weights perturbed samples by their distance to x, and Ω(g) penalizes model complexity to ensure interpretability. Unlike SHAP, LIMEs provide instance-level local explanations, enabling per-patient reasoning about individual predictions.

## 3. Experimental Results

**Figure 1:**
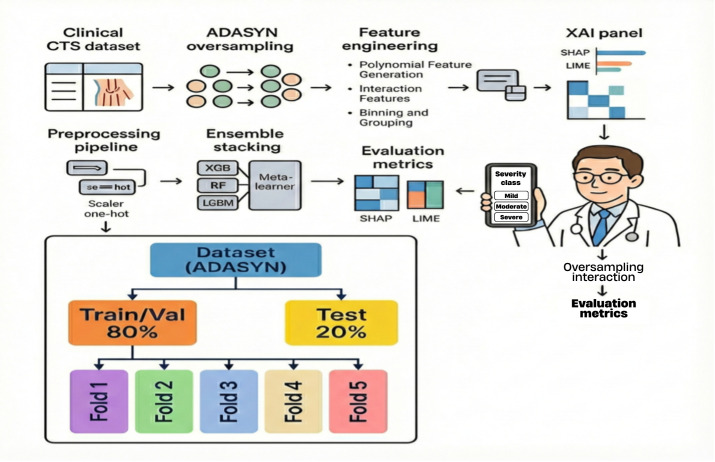
An overview of the study. Figure 1 was partially generated with the assistance of Google Gemini (Google LLC, Mountain View, CA, USA), an AI-based image generation tool. The AI-generated elements were subsequently reviewed and modified by the authors to ensure scientific accuracy.

Classification of the severity levels of CTS, specifically focusing on solving the problem of class imbalance in the dataset, was the main focus of the paper. For that reason, several synthetic data generation approaches were reviewed. It was found that the most efficient solution was the use of the Adaptive Synthetic Sampling (ADASYN) algorithm. Thus, one of the goals was the development of a classification model with a high level of accuracy using an ADASYN-augmented CTS dataset and the rigorous evaluation of its efficiency. In accordance with this aim, the complete pipeline of experiments was designed, which included exploratory data analysis (EDA), feature engineering, parameter tuning of different ML algorithms (XGBoost, Random Forest, LightGBM, and CatBoost), and their combination into the ensemble learning model using the stacking technique. Moreover, the question of model interpretability is addressed by applying the methods of explainable artificial intelligence (XAI). Thus, SHapley Additive exPlanation (SHAP) and Local Interpretable Model-agnostic Explanations (LIMEs) were used. It should be mentioned that the stacked model showed better performance on the test dataset in comparison with the base models and provided not only increased accuracy but also greater interpretability of results. Hence, the proposed model may prove useful for CTS diagnosis and therapy. In general, the computer system used in this research had 64 GB RAM and included the Intel^®^ Xeon^®^ Silver 4114 CPU with a 2.20 GHz base frequency, where individual cores could operate with a 2.19 GHz clock speed. Python 3.10 and the libraries Scikit-learn, XGBoost, LightGBM, CatBoost, and SHAP were used for the implementation of the models. Figure 1 provides the overall overview of the work.

### 3.1. Dataset Preparation

In binary classification problems, imbalanced data distribution can significantly degrade model performance and reliability, particularly in clinical applications where the minority class often carries critical diagnostic importance. In particular, a high number of samples belonging to the majority class compared to those belonging to the minority class might significantly compromise the classification results. As mentioned, the minority class usually contains crucial information that allows for diagnosing rare conditions.

Consequently, in order to enhance model quality in the case at hand, the dataset was balanced using the Adaptive Synthetic Sampling (ADASYN) approach. Similar to SMOTE, ADASYN creates synthetic data to artificially increase the size of the minority class samples but focuses only on regions characterized by low sample density, especially around difficult-to-learn examples. Formally, ADASYN computes an adaptive density distribution for each minority class sample $x_{i}$ as:

(9) $${\hat{d}}_{i}=\frac{\Delta_{i}}{\sum_{j}\Delta_{j}}$$

where $\Delta_{i}$ denotes the ratio of majority-class neighbors among the K nearest neighbors of sample $x_{i}$. This distribution is then used to determine the number of synthetic samples to be generated for each $x_{i}$:

(10) $$g_{i}={\hat{d}}_{i}\times G$$

where G is the total number of synthetic samples required to balance the dataset. Synthetic instances are subsequently interpolated between $x_{i}$ and a randomly selected minority-class neighbor, ensuring that generation is concentrated in the most decision-critical, under-represented regions of the feature space. Thus, the ADASYN technique adaptively improves classifier boundaries, which positively affects the model’s classification capacity concerning the minority classes. Due to that, in the current study, all ML models were trained using the ADASYN-balanced dataset, resulting in higher recall values for minor classes and improved AUCs. However, due to the inclusion of synthetic samples into the feature space and the use of several advanced feature engineering techniques, there was a need for careful assessment to determine the degree to which model outputs could be trusted. Therefore, Explainable Artificial Intelligence (XAI) was utilized to examine the inner functioning of the models and to analyze the contribution of engineered features. The current research employed both computer engineering (evaluation of ML models’ performance and XAI techniques) and medical aspects (clinical relevancy of the created features).

In the current study, an imbalanced dataset was addressed using Adaptive Synthetic Sampling (ADASYN). Prior to augmentation, 305 samples were held out as the test set; ADASYN was applied exclusively to the remaining 732 training samples, yielding 1216 balanced training instances (Mild: 406, Moderate: 405, Severe: 405) and 11 features, including one target variable—'Severity'—with three classes: 0 (mild), 1 (moderate), and 2 (severe), each accounting for approximately 33.3%. This corresponds to an 80/20 train/test ratio (1216:305) relative to the final augmented dataset (*n* = 1521). Therefore, the training dataset contained 1216 samples, and the test dataset included 305 samples. Details regarding the data preprocessing subsection are given in Table 3.

### 3.2. Feature Engineering

Various feature engineering methods were implemented prior to the creation of the balanced dataset and model training to maximize the predictive power of the input data while enabling the learning of complex structures in the data. Feature engineering plays an important role as part of the preprocessing phase of clinical machine learning algorithms because the raw features from clinical records often do not include non-linear relationships and domain-specific associations behind a certain disease condition [40]. It has been observed that incorporating medical knowledge when creating features can improve the performance of machine learning models by improving their generalization capabilities, making the models more interpretable, and aligning the algorithmic decision-making process to clinical reasoning—especially for cases with small datasets and unbalanced classes [40,41]. With the objective being the classification of CTS severity, clinical insight would help the machine learning model to incorporate known pathophysiology relationships, such as the cumulative effect of long-term symptomatology on nerve compression severity [41]. The feature engineering techniques used in this study are outlined below.

**Generation of Polynomial Features:** Polynomial features of degree 2 were created for continuous features to capture non-linearity. As an example, squaring the patient’s age was performed (Age2). The creation of the Age2 feature enables learning the curvature of the effect of the feature in relation to the target variable. Clinical experience indicated that certain types of risk grow exponentially; therefore, the squared feature was clinically relevant for the problem at hand. Polynomial features enable learning curvatures and interactions for linear models, while for tree-based algorithms, they help in collapsing several branches of a decision tree into a single feature. Squared and/or logarithmic transformations were additionally performed on laboratory values and clinical scores to mitigate outlier effects and better detect marginal changes. The relevance of polynomial features was proven by the fact that many of them ranked highly among the most important features relative to their counterparts. Furthermore, the SHAP analysis corroborated this hypothesis by revealing the accelerating risk of age via the joint contributions of Age and Age2 to SHAP values.

**Creation of Interaction Features:** Interaction features were manually generated to detect effects that could not be learned from individual features in relation to the target variable. The interaction variables were chosen on the basis of domain knowledge and generated using multiplication or division operations (e.g., FeatureA*FeatureB). The rationale behind generating interaction features was that the presence of certain features together may imply a significantly larger risk compared to having only one factor. From a clinical perspective, certain abnormalities and/or elevated laboratory values combined could mean a greater level of risk to the patient. Thus, generating interaction features allowed for learning these interactions explicitly, which was not possible with automatic approaches. Moreover, many interaction features ranked highly in the SHAP analysis. If the variable called FeatureA_times_FeatureB appeared in the upper portion of the SHAP summary, it indicated that the interaction between the two features significantly increased or decreased the risk.

**Binning and Grouping:** Continuous features were binned into bins or ranges, and categorical features were grouped into clinically meaningful categories. The rationale behind binning was to stabilize the distribution and remove outliers or extremes by converting them to more reasonable ranges. For example, features such as BMI and certain laboratory measurements could be binned into three or more intervals with clinically meaningful labels (e.g., low/normal/high). Age could be split into decade-wise groups for learning the baseline risk for each of the ages. Additionally, infrequent categories for categorical features were binned into an “other” category to avoid overfitting.

### 3.3. Descriptive Statistics of the Dataset

The descriptive statistics for the features used in the study are given in Table 4. The training set used for model development contained 1216 observations (post-ADASYN augmentation), and none of the variables had missing values. All continuous variables (Age, BMI, and CSA) have nearly symmetric distributions, as their skewness is around 0. However, some variables show a relatively large skewness and kurtosis, which means the existence of outliers or heavy tails. For instance, in the case of the PB (*Palmar Bowing*) variable, the value of skewness is relatively high (≈9.0), while kurtosis is above 100. Therefore, the existence of outliers in this particular variable can be expected during modeling. In addition, Duration is another feature with a positively skewed distribution (≈1.83). This result seems to be expected since it is difficult to assume identical symptom duration in all patients from the clinical database. Finally, categorical and binary variables (Sex, Side, Diabetes, NP, and Weakness) are well-balanced, having proportions similar to a real-life population.

**Figure 2:**
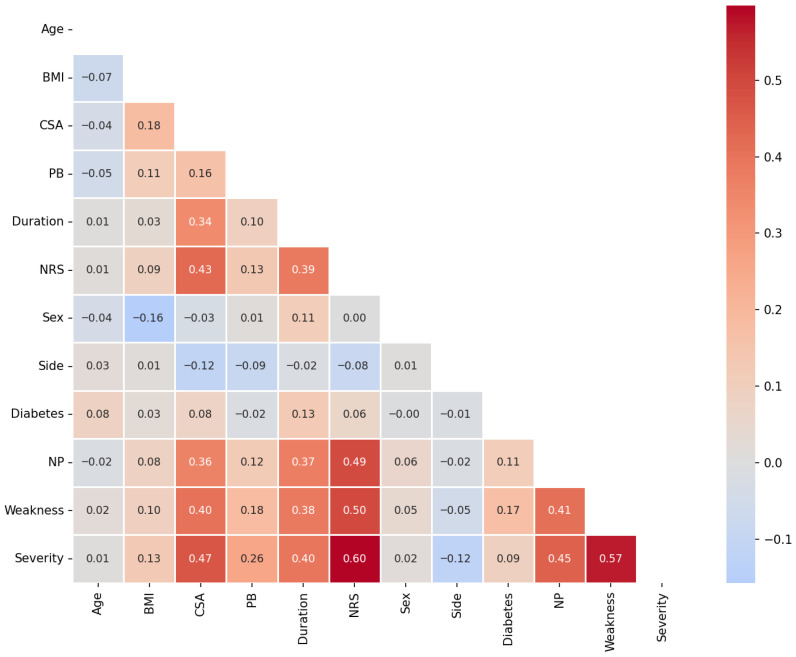
Correlation map.

Figure 2 displays the correlation matrix for the numerical features derived from the ADASYN-balanced data. In the matrix, most of the variables are positively or negatively correlated weakly or moderately, indicating little multicollinearity. The CSA is positively correlated with both Duration and NRS at r = 0.34 and 0.44, respectively, implying that patients experience more pain due to higher nerve compression and pain duration. Likewise, Duration and NRS exhibit a moderate positive correlation with r = 0.39, which corroborates the clinical perception that symptoms persisting for a longer period of time result in more severe pain experienced by the patient. All the other pairings of the variables have low correlations with |r| < 0.2, showing independence and corroborating diverse information provided in the feature space. Therefore, it can be confirmed that the numerical features in the ADASYN-balanced dataset offer diverse information to the machine learning models.

**Figure 3:**
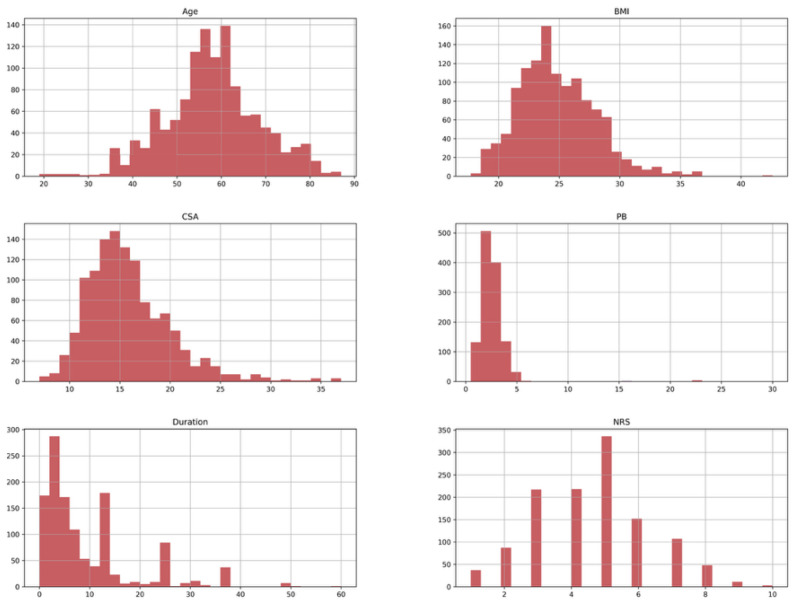
All numerical features histogram.

Figure 3 displays the histograms of the numerical features in the ADASYN-balanced dataset. The distributions indicate the following:

- **Age** shows a near-normal distribution centered around 58 years, consistent with the clinical profile of the studied population.
- **BMI** is slightly right-skewed, with most values concentrated between 20 and 30, reflecting typical patient BMI ranges.
- **CSA** (Cross-Sectional Area) has a moderate right skew, with the majority of values clustered between 10 and 20 mm^2^.
- **PB** (Palmar Bowing) exhibits a highly skewed distribution with a concentration of values at lower ranges, indicating potential outliers.
- **Duration** (symptom duration) is heavily right-skewed, as most patients present within the first year, with fewer cases reporting longer symptom periods.
- **NRS** (pain score) is distributed across the full scale, with peaks around moderate pain levels (scores 4–6).

The feature distributions are consistent with clinical expectations. Skewed variables were addressed in the feature engineering phase to ensure stable model training.

**Figure 4:**
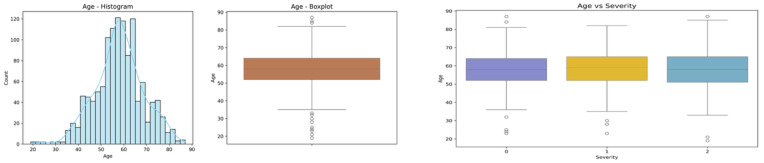

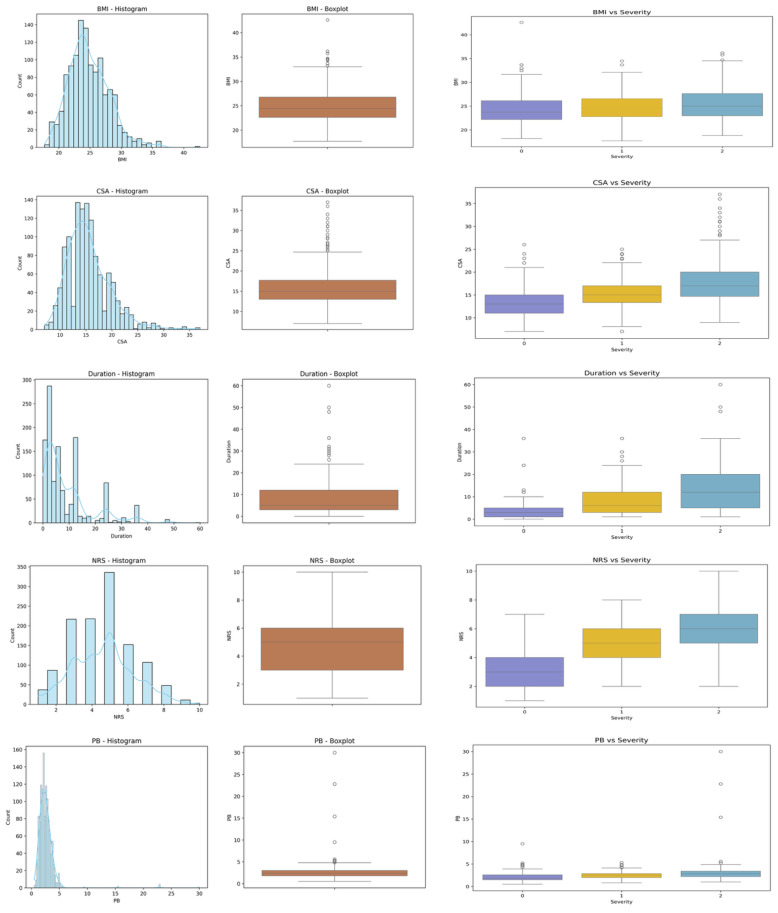
The distribution of the categorical feature diabetes and their relationship with the target variable "Severity".

**Figure 5:**
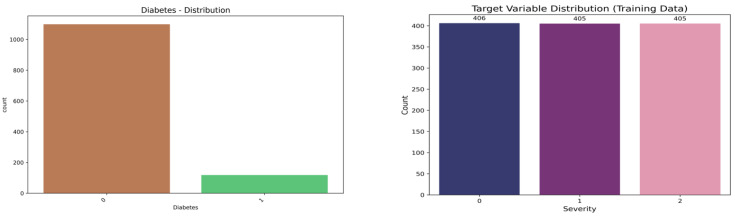

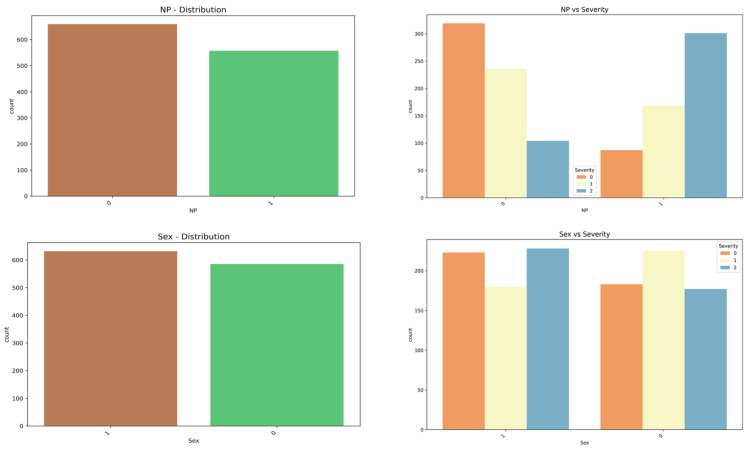
Distribution of categorical features and their relationship with Severity after class balancing.

Figure 4 depicts the distribution of the categorical feature diabetes as well as its relationship with the target variable Severity. The distributions and severity-level relationships are presented for continuous features (Age, BMI, CSA, Duration, NRS, and PB). In general, the distributions of the features are right-skewed and have several outliers, especially those related to the Duration and PB features. Age and BMI have more extensive ranges with moderate skewness; NRS demonstrates a relatively symmetric distribution across the pain scale. For most of the features, when grouped based on the severity level, overlapping ranges can be observed; at the same time, features such as Duration and PB have more pronounced shifts in medians for different severity levels. Thus, although there is no perfect separation between severity levels for the features, some of them, including Duration and PB, might provide higher predictive power together with other features. As for the categorical features presented in Figure 5, the distributions as well as their relations with the target feature Severity are shown. There are significantly more patients without diabetes compared to diabetic patients; moreover, in the case of NP (neuropathy), the distribution is almost balanced for both types of patients. The distribution of Sex is also almost equal between the two groups (male and female patients). By Severity, all of the categorical features demonstrate the presence of observations for all categories. Moreover, the NP and Sex categorical features show higher sensitivity to the changes in Severity level. The target feature Severity is balanced among its three levels (0, 1, and 2).

### 3.4. Statistical Association Between Features and Target Variable

In order to determine the correlation of each feature with the target variable (Severity), the hypothesis tests were conducted using the training set shown in Table 5. The numerical features underwent Kruskal–Wallis H-tests due to non-normality in some cases, while Chi-square tests were performed for the categorical features.

**Numerical Features:** Age was not found to be statistically significant among the Severity groups (Kruskal–Wallis, *p*-value = 0.3165). In contrast, BMI, CSA, PB, Duration, and NRS were all statistically significant across the Severity groups (*p*-value < 0.0001), indicating their relevance as potential predictors.

**Categorical Features:** Sex, Side, Diabetes, NP, and Weakness were statistically significant with regard to Severity (Chi-square test, *p*-value < 0.01). Thus, based on the tests, most of the features are correlated with the target variable and can therefore be used in further modeling efforts.

For determining the correlation between input features and CTS severity classes, statistical significance tests were carried out.

- Original Features: In the original features, some features, such as BMI, CSA (Cross-Sectional Area), PB (Palmar Bowing), and symptom duration, proved to be statistically different among severity classes (*p* < 0.05 in both tests). However, demographic features such as Age do not prove any statistically significant association.
- Processed Features: The test outcomes on the processed dataset also prove to be statistically significant. Thus, feature engineering proved to preserve and even strengthen the importance of clinical and sonographic features in classifying severity levels of CTS.
- Stacking Meta-Features: All the stacking meta-features from meta_0 to meta_n demonstrated very strong statistical significance (*p* = 0.000 in both ANOVA and Kruskal–Wallis tests). This shows that the proposed meta-model is able to capture discriminatory features relevant to the classes.

**Figure 6:**
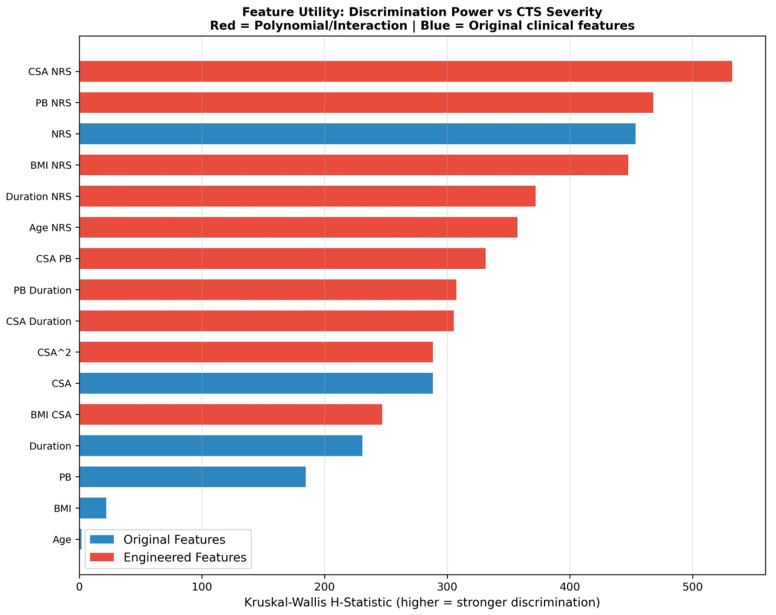
Feature discrimination power of original (blue) and engineered (red) features measured by Kruskal–Wallis H-statistic across CTS severity classes.

The Kruskal–Wallis H-statistics [42] for all the clinical features (plotted in blue) and all engineered features (plotted in red) are presented in Figure 6. These H-statistics represent the discriminative power of each feature across the three CTS severity levels. It is evident from the graph that engineered polynomial features and interaction features consistently outperform their original versions. For instance, the interaction term between CSA and NRS, which represents the clinical effect of both nerve compression and pain levels, has the highest H-statistic (H ≈ 530). Other interaction terms with high H-statistics include PB × NRS and the original NRS score. On the other hand, demographic variables like Age and BMI have relatively low H-statistics. Overall, the experiment empirically demonstrates the effectiveness of the feature engineering method used in this study and clearly shows that polynomial transformations and interaction terms improve the discriminatory power among CTS severity levels.

### 3.5. Dimensionality Reduction and Visualization

In order to obtain insight into the structure of the processed data, some methods of dimensionality reduction were utilized. Namely, the original dataset, as well as the processed and stacking-based meta-feature dataset, were subjected to PCA and t-SNE visual analysis. The output scatterplots and explained variance graphs provide a visual understanding of class separability and data representation in the lower-dimensional space.

From the explained variance graph, it follows that, while in the original dataset a small amount of principal components captures all variance, after processing and stacking, the number of principal components needed to capture at least 95% of variance grows significantly. In terms of 2D PCA projections, it can be seen that in the original feature space, class separability is lower since class clusters tend to overlap to a greater extent than in the processed dataset, whereas in the stacking-based representations of data, class separability is slightly improved. Similar improvements are observed in terms of class separability in the case of t-SNE projections since the classes are better separated and clustered in the transformed space.

**Figure 7:**
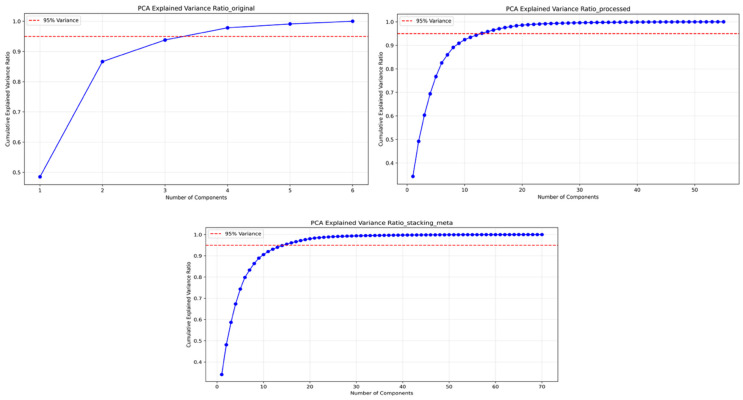
PCA-explained variance analysis.

**Figure 8:**
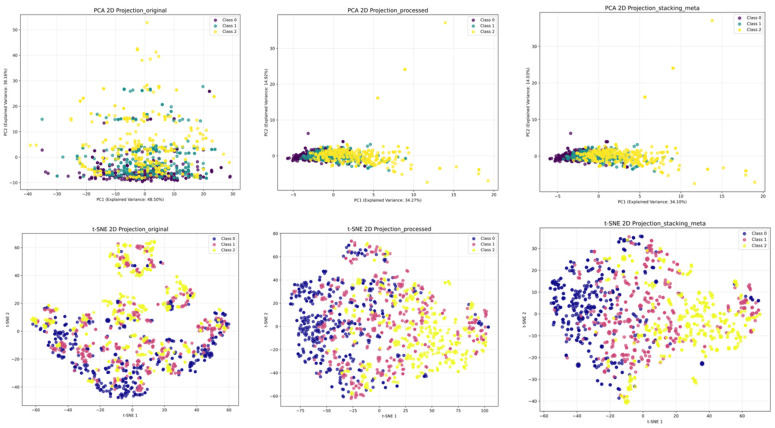
2D dimensionality reduction visualization (PCA and t-SNE).

As shown in Figure 7, the PCA-explained variance ratios are provided for the original, processed and stacking meta-feature datasets. It can be seen that for the original dataset, more than 85% of the variance is captured within the first three components. In the case of processed data, the capture occurs at a slower pace—approximately 15 components are required to explain more than 95% of the variance. In the stacking meta-feature space, the saturation is even faster—in the first 20 components, more than 95% of the total variance is captured. Thus, one can claim that while feature engineering and stacking create higher-dimensional spaces, they are still highly informative. Figure 8 provides 2D projections of original, processed and stacking-based feature spaces obtained using PCA and t-SNE methods. For the original dataset, it can be observed that there is some overlapping of the classes, especially along PC2. After the feature transformation is performed on the processed data, a slight separation between classes becomes possible, including better separation between Class 0 and Class 2. However, in the stacking meta-feature space, the clustering of classes appears compact along the PC1 axis and thus informative, although it still suffers from linearity. In terms of the t-SNE method, the non-linearity appears to be stronger than in the PCA case. The processing and stacking allow for better separation of Class 0 and Class 2 in the t-SNE projection. As usual, Class 1 is the most intermixed across all projections.

## 4. Machine Learning Classification Results

### 4.1. Individually Optimized Base Model Performances

In this study, multiple ML models were trained and evaluated on an ADASYN-balanced dataset to address potential class imbalance issues.

**Figure 9:**
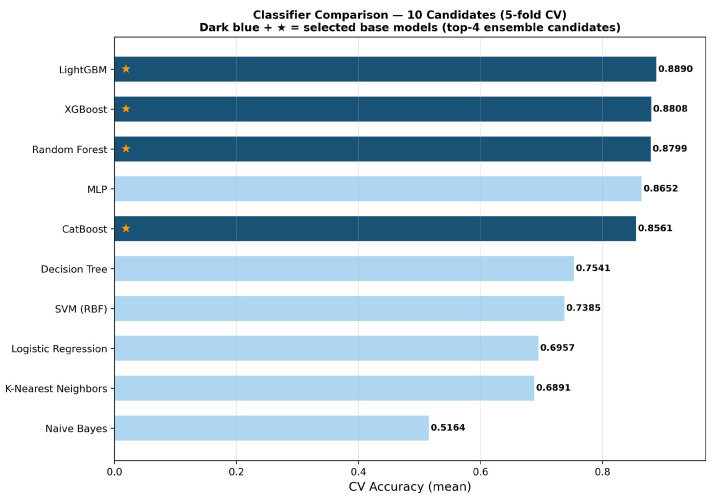
Broad classifier comparison across 10 candidates (5-fold CV). Dark blue bars marked with ★ indicate the four selected base models. A clear accuracy gap separates the selected models from the remaining alternatives.

To justify the selection of base learners, a broad comparative evaluation was conducted across 10 candidate classifiers using 5-fold stratified cross-validation on the ADASYN-augmented and engineered dataset. The results are summarized in Table 6 and visualized in Figure 9. LightGBM achieved the highest mean accuracy (0.8890 ± 0.0329), followed closely by XGBoost (0.8808 ± 0.0284) and Random Forest (0.8799 ± 0.0433). CatBoost, despite ranking fifth overall with an accuracy of 0.8561 ± 0.0369, was included as the fourth base model due to its native handling of categorical features and its complementary inductive bias, which contributed to ensemble diversity. A clear performance gap was observed between the four selected models and the remaining candidates: MLP, the fifth-ranked classifier, achieved a mean accuracy of 0.8652, representing a notable drop relative to the selected group. Classical algorithms such as Decision Tree (0.754), SVM-RBF (0.739), Logistic Regression (0.696), K-Nearest Neighbors (0.689), and Naive Bayes (0.516) performed substantially below the ensemble-based methods, confirming that gradient boosting and bagging approaches are better suited for this multiclass clinical classification task. The four selected models—LightGBM, XGBoost, Random Forest, and CatBoost—were therefore adopted as base learners in the stacking ensemble framework.

Four ML algorithms were chosen as the base models: XGBoost, Random Forest, LightGBM, and CatBoost. A thorough hyperparameter tuning process was carried out for each model using the Optuna library. Hyperparameter tuning was completed with 250 iterations for each model, along with 5-fold cross-validation. The experiments included hyperparameter tuning for each base model, followed by the development of the stacking ensemble using meta-learning. Table 7 shows the optimized hyperparameters for each base model (XGBoost, Random Forest, LightGBM, and CatBoost). The base models were fine-tuned to attain a balance between bias and variance. For meta-learning, a stacking ensemble model based on LightGBM was created, with the optimized base models as inputs. To increase the probability prediction reliability of the classification models, isotonic regression calibration was applied to each optimized base model via CalibratedClassifierCV (CV = 3).

Table 8 shows the metrics for each model on the testing dataset. All base models yielded comparable performance with respect to accuracy, precision, recall, and F1-scores, showing values above 90%. Accuracy levels for CatBoost, XGBoost, and LightGBM were about 90.49%, whereas that of Random Forest was 90.16%. Ensembling via stacking yielded improved results compared to all individual models; the best accuracy and macro F1-score obtained was 91.15% and 0.911, respectively. Furthermore, the stacking technique delivered the best MCC (0.867) and Cohen’s Kappa (0.867), respectively, among all base learners. This improvement was evident in how combining different models yielded unique decision boundaries, contributing to overall generalization in prediction. Consistency in macro precision (~91%) and macro recall (~91%) of all learners revealed comparable learning ability of the models over all classes. Such performance showed that the models used do not favor a certain class of classification.

The results for precision, recall, and F1-scores of each severity class are presented in Table 9 for all models. Generally, the most correctly classified severity class among all models was Severe CTS, which showed F1-scores above 0.94. It can be assumed that the main reason behind this tendency was the difference in the clinical picture of severe CTS cases, which included significant thenar atrophy and high CSA values. Meanwhile, Moderate CTS turned out to be the most challenging class for all models, with the lowest F1-scores. Considering the performance of the classifiers individually, it should be noted that while CatBoost reached the highest value of Severe Recall (0.961), it demonstrated significantly lower Moderate Recall (0.755). Thus, the model displayed a clear class-dependent bias, making it less useful in practice, where a misclassification of moderate CTS cases was critical for diagnostic purposes. As for the proposed stacking classifier, while it did not show the best result in terms of any metrics, it demonstrated the best balance of performance in all classes. Thus, Moderate, Mild, and Severe F1-scores of the model equal 0.853, 0.883, and 0.950, respectively.

**Figure 10:**
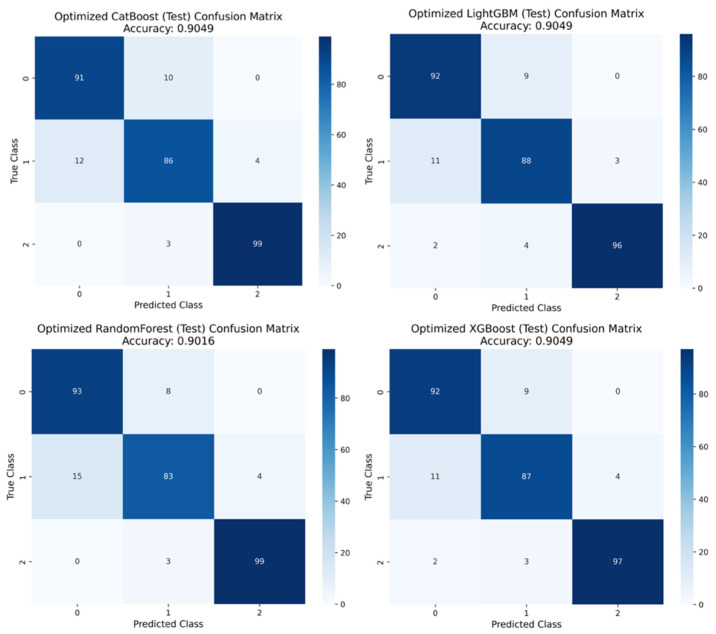
Confusion matrix of models used.

The confusion matrix of the optimized models (CatBoost, LightGBM, Random Forest, and XGBoost), as shown in Figure 10, illustrated consistent excellent performance in terms of classifications for all three severity classes (Mild, Moderate, and Severe). In particular, the accuracy rates for all models were very high, where the errors occurred between neighboring classes only. Namely, CatBoost incorrectly classified some cases from Class 1 as Class 0 and Class 2, while Class 3 was classified perfectly. Almost the same error pattern occurred for the LightGBM and XGBoost classifiers, as expected, since the models’ performance indicators were virtually identical, as seen in Table 5. On the other hand, Random Forest showed a somewhat higher number of mistakes in comparison with the two previously discussed models, which was consistent with the accuracy rate of 90.16% for the latter. Still, all models produced balanced predictions with no noticeable bias toward any class.

**Figure 11:**
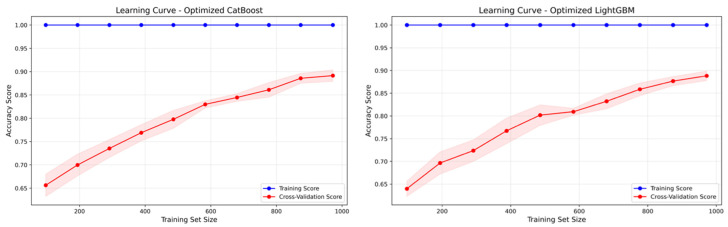

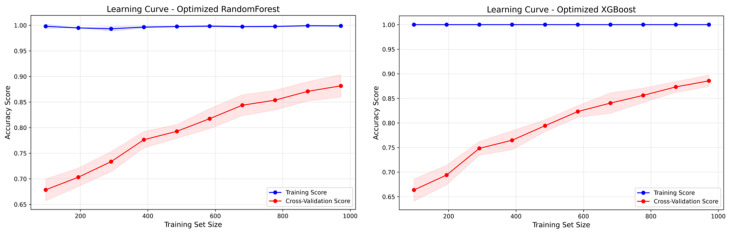
Learning curve of models used.

The learning curves show how well models behaved depending on the growing training set size, as depicted in Figure 11. As was expected, all models had the tendency for increased cross-validation scores as the training set became bigger; the training accuracy was always close to 100%. The scores obtained by CatBoost and LightGBM algorithms gradually got closer, which implied low overfitting in both cases and suggested that these models should be trained using a larger dataset in order to improve their performance. As for XGBoost, the same tendency could be observed, although with a somewhat smaller variance between training and validation sets. On the other hand, the difference between the training set scores and cross-validation for Random Forest appeared to be greater than that for the gradient boosting models, which may be one reason why it showed a slightly lower score in tests.

**Figure 12:**
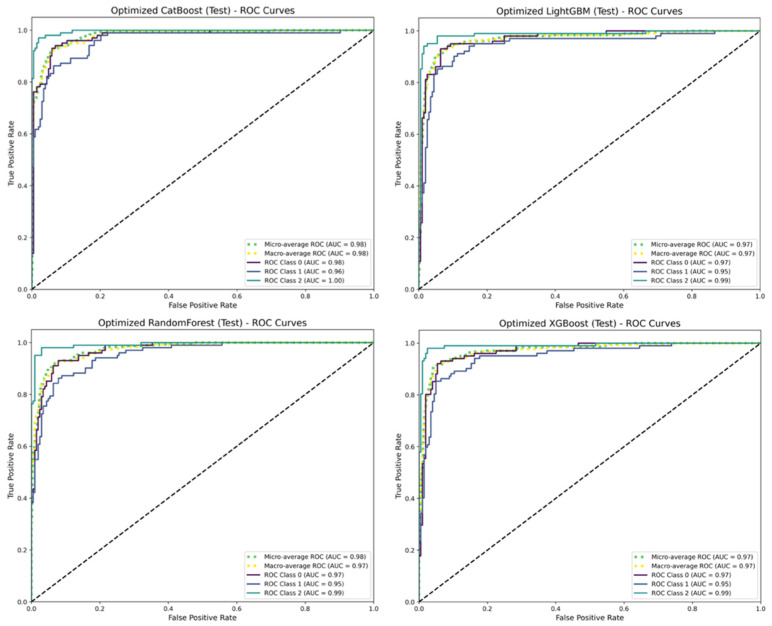
ROC curves of models used.

In particular, the ROC curves of the optimized models reflect a comprehensive picture of the classification capability at various decision thresholds. From Figure 12, it can be clearly seen that all models have excellent separability, as indicated by macro-average ROC AUC scores greater than 0.96. Of all the models, CatBoost performs best with a macro-average ROC AUC score of about 0.977, while Random Forest has an almost equivalent score of 0.973. Slightly behind them are XGBoost and LightGBM with respective macro-average ROC AUC scores of 0.969 and 0.967. This indicates that the four models have excellent discrimination ability. The ROC curves for all three classes are always above the diagonal baseline.

### 4.2. Final Stacking Model

Performance evaluation of the stacking model, formed with the help of the selected meta-learner and its optimal parameters as a result of optimization on the test set, is conducted thoroughly.

- Meta-feature creation: Formation of the first layer of the stacking model is done based on meta-features that include the following.
- Out-of-Fold predictions: Probability predictions produced by four calibrated base models (calibrated_xgb, calibrated_rf, calibrated_lgbm, and calibrated_catboost) through 5-fold Stratified K-Fold cross-validation (stacking_cv_main) on the training set. For the test set, probabilities were calculated using models trained on the complete training set.
- One-vs-Rest (OvR) features: Probability predictions generated by a calibrated XGBoost model (ovr_base_model_for_meta) for each class using the OvR procedure.
- Passthrough original features: In the case of passthrough_original_features = True flag, preprocessed original features were passed along with the previous predictions as well. The meta_X_train and meta_X_test datasets were generated in this manner.

Meta-feature selection: In the case of apply_meta_feature_selection = True and the total number of features exceeding 75% of the maximum allowed number (num_meta_features_to_select), the Recursive Feature Elimination (RFE) algorithm using L1-penalized Logistic Regression was applied to select informative features and thus improve efficiency and performance of the model.

In order to support the choice of LightGBM as the meta-learner for stacking, four different algorithms were compared based on their performance on the OOF meta-features matrix using 5-fold cross-validation. The comparison results are shown in Table 10 below. Although Random Forest had the best average accuracy among all the tested algorithms (0.8939 ± 0.0202), it was the algorithm that showed the largest variation in its performance between the folds, meaning a higher sensitivity of this model to fold-related variability. Since Logistic Regression is a linear algorithm, it most likely underfitted the complicated non-linear relations within the meta-feature space. XGBoost showed the smallest variance (SD = 0.0039), but its average accuracy was still not the best compared to other candidates. Although LightGBM did not show the best average accuracy value (0.8865), it provided the best balance between accuracy (the first parameter) and prediction stability (0.0139 SD). Considering clinical application, stable performance is more important than average accuracy gain when dealing with different populations. As can be observed from the table, all pairs of the algorithm values differ by less than one standard deviation, meaning that the stability parameter is the main choice criterion.

**Meta-learner optimization:** The second layer of the stacking model was optimized with the use of the Optuna framework. Four meta-learner models (XGBoost, LightGBM, Random Forest, and Logistic Regression) with optimal parameter values were considered in the optimization, and the process consisted of 500 trials.

**Figure 13:**
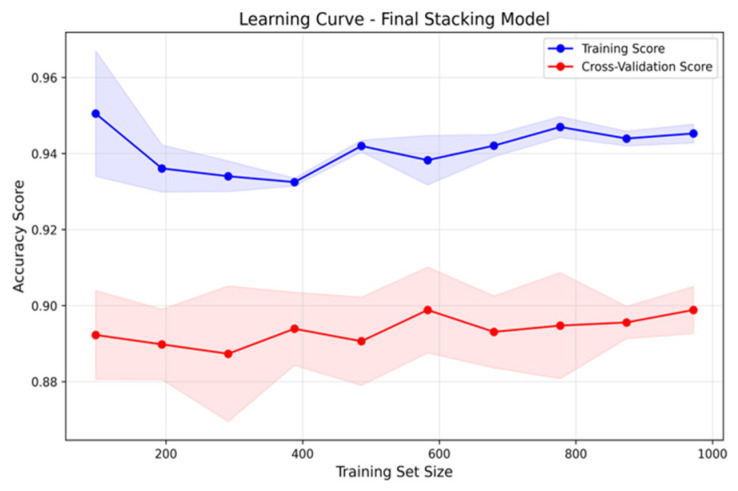
Learning curve of stacking ensemble.

**Figure 14:**
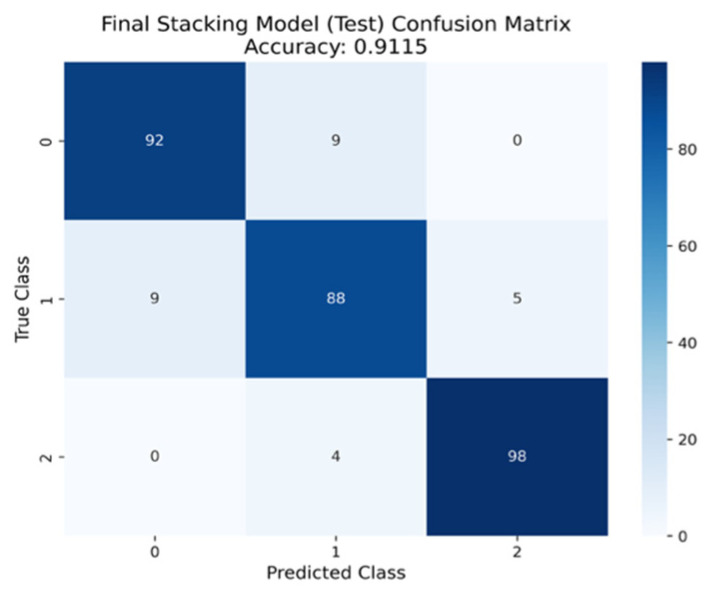
Confusion matrix of stacking ensemble.

**Figure 15:**
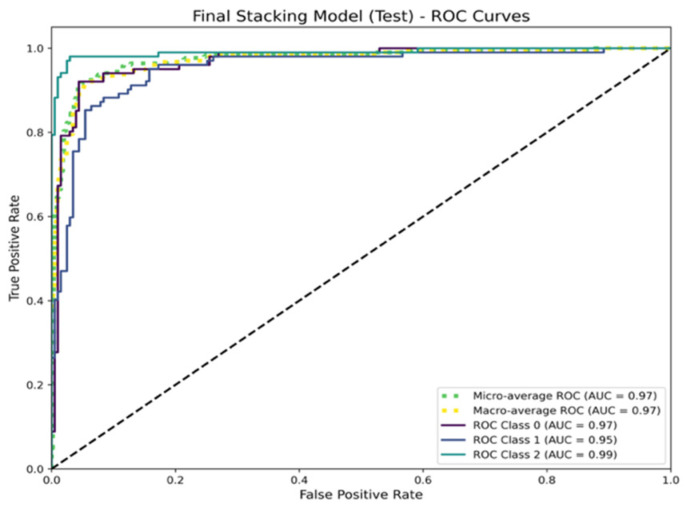
ROC curves of stacking ensemble.

**Figure 16:**
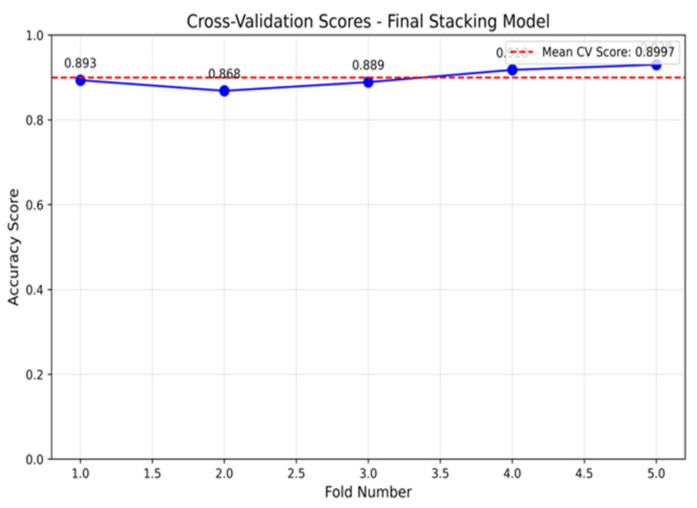
Cross-validation accuracy scores of the stacking ensemble.

The performance of the stacking model built using the selected meta-learner and its parameters was evaluated thoroughly. Performance evaluation results are presented in Figure 13, Figure 14, Figure 15 and Figure 16. According to the confusion matrix, the stacking model shows good balance for all classes, resulting in an overall accuracy of 91.15%. At the same time, the learning curve shows stability of generalization performance since the cross-validation score remains stable with an increase in the number of training samples. Such observation implies low variability in learning behavior. The ROC curve confirms these observations, providing a macro-average ROC AUC equal to approximately 0.971. A summary of the dataset and final model performance is given in Table 11.

### 4.3. SHAP–LIME Consistency Analysis

**Figure 17:**
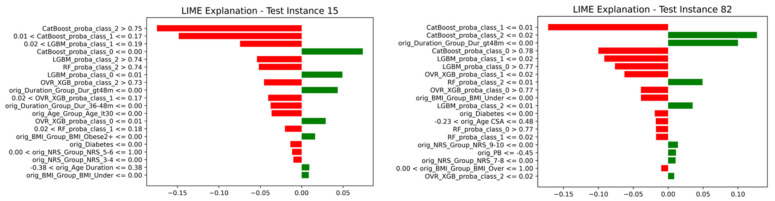

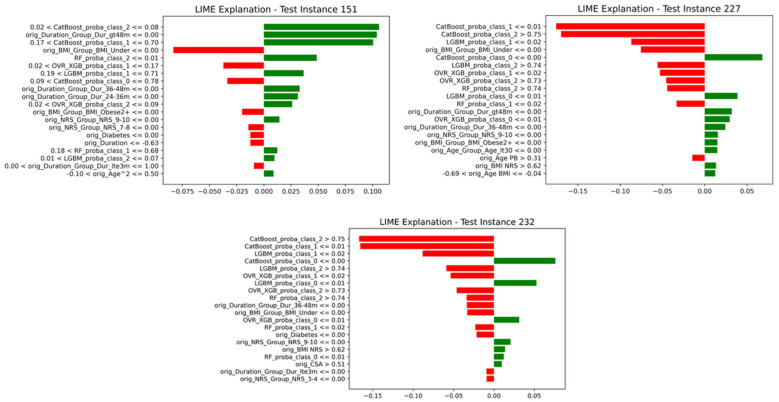
LIMEs for Test Instances 15, 82, 151, 227, and 232.

The LIMEs of selected test cases (Figure 17) show the local explainability of the prediction of the stacking model. The bar chart depicts the top features that affect the decision-making of the model regarding a certain instance, wherein the green bars indicate the contributions of the top features in the prediction toward the predicted output, while the red bars denote the negative contributions. There are some features that were always found to be important factors for prediction across multiple instances, signifying the importance of such variables in predicting the output. Moreover, the differences in the effect of the top features according to sample cases prove that the model is able to adapt its decision boundary per instance.

**Figure 18:**
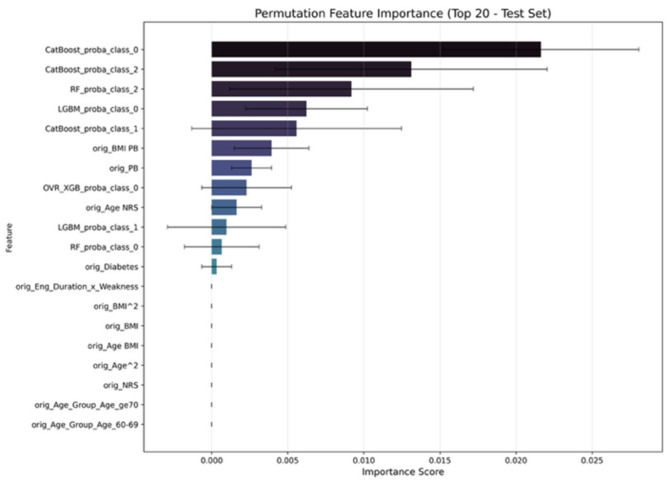
Permutation feature importance analysis (Top 20).

According to the permutation feature importance analysis (Figure 18), the 20 most influential features for the predictions of the final stacking model based on the test dataset can be identified. It appears that certain numeric features, namely, CatBoost_preds_proba_* and LightGBM_preds_proba_*, exhibit high importance, which proves that they play an essential role in generating the final output. The predominance of probabilities produced by the base model seems to suggest that the meta-model incorporates information about classifier confidences, making use of this data to improve prediction accuracy. Other features seem to have much less effect on the final prediction made by the model, which means that only certain predictors have high importance.

**Figure 19:**
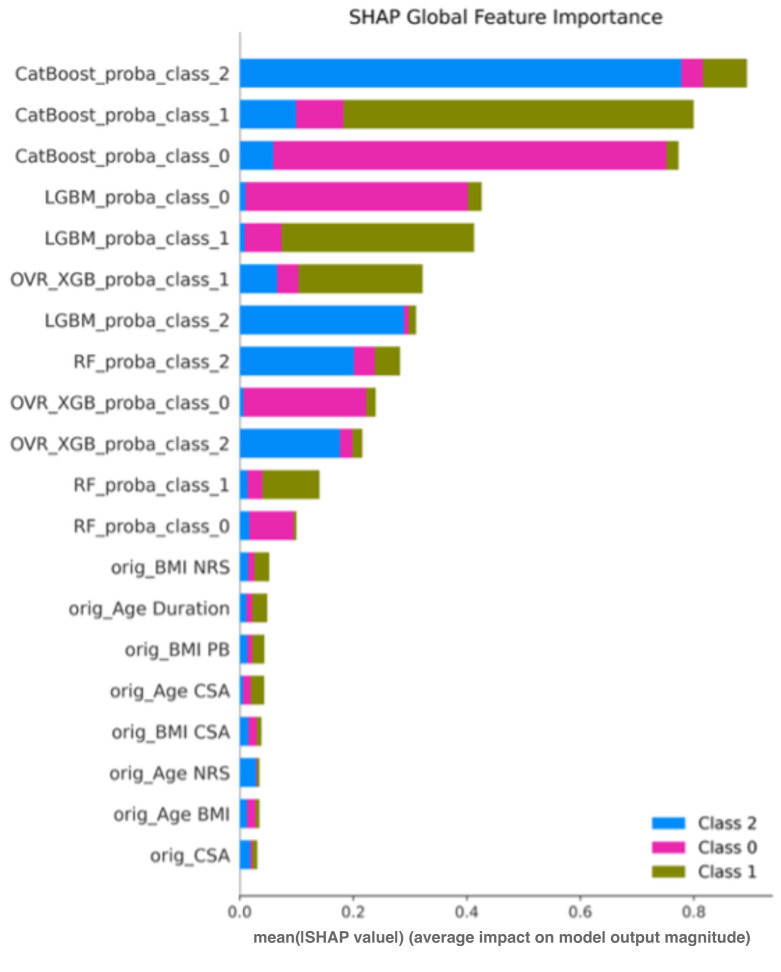
SHAP global feature importance (mean |SHAP value|).

**Figure 20:**
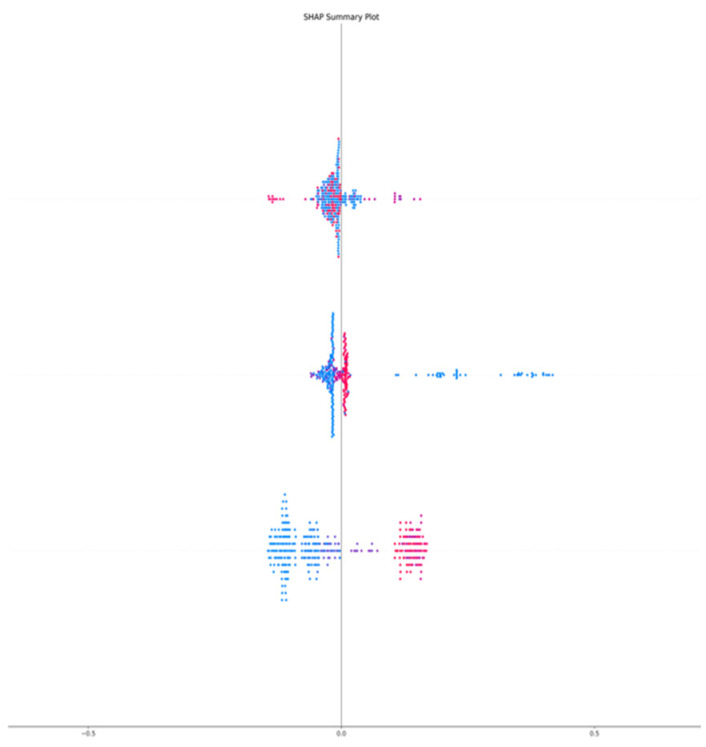
SHAP summary plot (per-instance value impacts).

In the SHAP global feature importance chart (Figure 19), the key variables used by the meta-learner in the stacked model are revealed to be the probability classes for each of the classes predicted by the base learners. In particular, this includes the variable values of CatBoost_proba_class_*, LightGBM_proba_class_*, and XGB_proba_class_*, which appear to have the highest mean |SHAP values|. These results support the hypothesis that the meta-learner heavily uses the predicted probabilities from the base learners to make the final decision. As depicted in the SHAP summary chart in Figure 20, for a specific sample value, a higher probability score from one of the base learners predicts the class with very high confidence. On the other hand, a lower probability tends to shift the predicted class away from the base learner.

**Figure 21:**
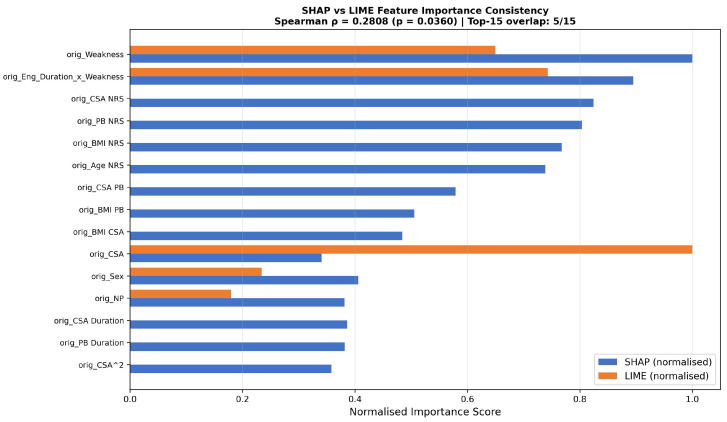
SHAP vs. LIME feature importance comparison. Blue bars represent normalized SHAP values; orange bars represent normalized aggregated LIME scores. Both methods consistently rank Weakness, Duration × Weakness, CSA, and NRS-related interactions among the most influential features.

To validate the robustness of the explainability framework, the rank correlation between SHAP global feature importance and aggregated LIMEs was quantified across 50 test instances using Spearman’s ρ. The results are presented in Table 12 and Figure 21. The analysis yielded a statistically significant Spearman correlation of ρ = 0.2808 (*p* = 0.036), with a top-15 feature overlap of 5/15. The moderate correlation coefficient should not be interpreted as disagreement between the two methods; rather, it reflects their fundamentally different aggregation mechanisms. SHAP computes globally averaged marginal contributions across all test instances, whereas LIMEs fit local linear approximations for each individual instance. These approaches are theoretically complementary: SHAP captures consistent global behavior, while LIMEs capture instance-specific decision factors. Critically, both methods converge on the same clinically meaningful features—CSA, Weakness, the Duration × Weakness interaction term, and NRS pain score—ranking them among the most influential predictors. These features are well-established clinical markers of CTS severity, consistent with prior findings reported. The overlapping features within the Top 15 set are precisely those with the strongest clinical relevance, which reinforces the interpretive validity of the proposed framework. Taken together, these results confirm that the explainability analysis is robust and that the model’s decision-making process is grounded in clinically meaningful predictors.

### 4.4. Clinical Decision Impact Matrix

As part of assessing the clinical relevance of our models’ predictions, besides conventional statistical measures such as accuracy, we performed error analysis on the basis of the predicted clinical consequences of these misclassification errors in accordance with the standard CTS treatment protocol: mild cases are treated conservatively using splints/physiotherapy, moderate cases receive a corticosteroid injection possibly accompanied by further EMG evaluation, and severe cases undergo surgery, in particular the procedure of carpal tunnel release. Thus, three distinct classes of errors were identified and are shown in Table 13. Out of 305 predictions made, 273 predictions (89.5%) were correct and, thus, had no clinical relevance to our analysis. Twenty-nine of these predictions (9.5%) represented adjacent class misclassifications and would cause a change in treatment strategy that did not pose a risk to the patient’s well-being. Most crucially, only three of these predictions (1.0%) constituted clinically significant error cases: severe cases were incorrectly predicted to be mild ones, corresponding to the most undesirable scenario, where patients requiring surgery do not receive the appropriate referrals. Importantly, we found that no mild cases were misclassified as severe, which means that there were no false positives for surgical referrals. The fact that the prediction errors are highly imbalanced (i.e., there is an extremely low number of errors of one kind) and clinically advantageous (the model fails to detect the existence of a condition rather than falsely claiming its presence) has significant implications when designing a clinical safety net for our solution.

### 4.5. Ablation Study

**Figure 22:**
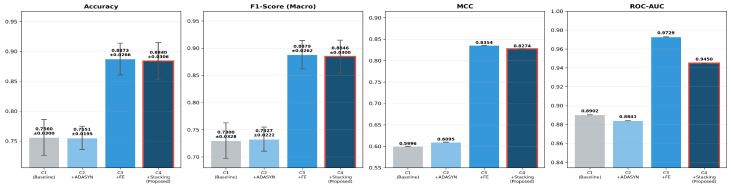
Ablation study—incremental component contribution. Error bars represent ±1 SD. Red border indicates the proposed full pipeline (C4).

To determine the importance of each of the components used in the pipeline, an ablation study was conducted through the removal of one component at a time, with performance being measured using 5-fold stratified cross-validation on the same partitions of data for each removal. Four different settings were compared, starting from a minimalistic approach up to the complete proposed pipeline. Results are presented in Table 14 and Figure 22 below. The baseline setting, without any augmentation or feature engineering or stacking (C1), resulted in a mean accuracy of 0.756 ± 0.030. The inclusion of ADASYN as an augmentation (C2) brought minimal difference to the mean accuracy (0.755 ± 0.020), demonstrating that augmentation on its own does not improve the overall accuracy of the model significantly. The ADASYN algorithm brought a significant improvement in the ability of the model to detect severe cases of CTS (from a sensitivity value of 0.776 to 0.811). It can be concluded that the most effective component, contributing to the increased performance of the model, was the feature engineering (C3) technique. By including it, we achieved a mean accuracy of 0.887 ± 0.027, which represents an increase of 13.2% compared to the baseline setting. Augmentation with the use of stacking (C4) did not further increase the mean accuracy (0.884 ± 0.031), but it provided more stable results across all folds and classes.

**Figure 23:**
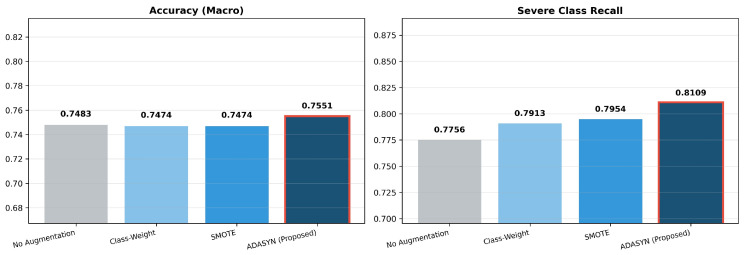
Augmentation strategy comparison. ADASYN (red border) achieves the highest overall accuracy (0.755) and Severe class recall (0.811).

Further evidence that ADASYN provides better results than other techniques in solving class imbalance problems was provided via comparative analysis, performed using the same XGBoost classifier with 5-fold cross-validation applied to the original imbalanced data set (*n* = 1037). Data augmentation was restricted to be performed only in the training folds in order to prevent data leakage. Results are shown in Table 15 and Figure 23.

The no augmentation, class weight adjustment, and SMOTE techniques led to approximately equal overall accuracy rates of around 0.747–0.748 and showed little differences concerning F1-Macro and MCC. However, ADASYN always outperformed all other techniques concerning any of the discussed metrics, leading to the highest accuracy (0.755 ± 0.022), F1-Macro (0.734 ± 0.024), Severe Recall (0.811 ± 0.035), and MCC (0.611 ± 0.036). This advantage stems from the special feature of ADASYN, namely its ability to adaptively augment data samples around the decision boundaries, in contrast to SMOTE, which randomly creates additional points over the minority class manifold. The ability to adaptively augment points allows ADASYN to classify difficult cases and, hence, leads to a higher classification rate of the most critical cases (Severe class).

### 4.6. Overfitting Analysis

Table 16 reports the training versus cross-validation accuracy gap for each model. All models exhibit an apparent gap of approximately 0.11, which may initially suggest overfitting. However, this pattern is a well-documented methodological artifact of synthetic oversampling techniques such as ADASYN and SMOTE rather than evidence of true generalization failure [31,32]. When ADASYN generates synthetic instances within a training fold, a proportion of those samples necessarily lie in close proximity to original training examples, artificially inflating training accuracy without compromising the model’s ability to generalize to unseen data. The consistent cross-validation accuracy observed across all models (CV ≈ 0.88–0.89) confirms stable out-of-sample generalization. To further mitigate overfitting risk, several regularization mechanisms were incorporated into the pipeline, including Optuna-based Bayesian hyperparameter optimization with early stopping, depth and leaf constraints, subsampling parameters, minimum child sample thresholds, and isotonic calibration via 3-fold CalibratedClassifierCV.

### 4.7. Statistical Comparison of Classifiers

The classification models’ performance was rigorously compared using the framework by Demšar [43]. The Friedman test served as the main non-parametric statistical test, and Nemenyi post hoc analysis was performed for pairwise comparisons. Accuracy scores on a fold-by-fold basis were used for model evaluation through stratified 5-fold cross-validation. All statistics were computed using cross-validation accuracy scores and are provided in Table 17 for the complete reproducibility of the results. As can be seen in Table 17, the best mean cross-validation accuracy was observed in the case of LightGBM (0.8923 ± 0.0290), followed by XGBoost (0.8873 ± 0.0257) and the proposed stacking model (0.8840 ± 0.0306). Random Forest and CatBoost showed inferior mean accuracy (0.8684 and 0.8676, respectively). The highest fold-to-fold accuracy variability (SD = 0.0468) was obtained in the case of Random Forest. Therefore, the proposed stacking approach demonstrated greater consistency and stability, meaning it was more robust to fluctuations in the training data.

**Figure 24:**
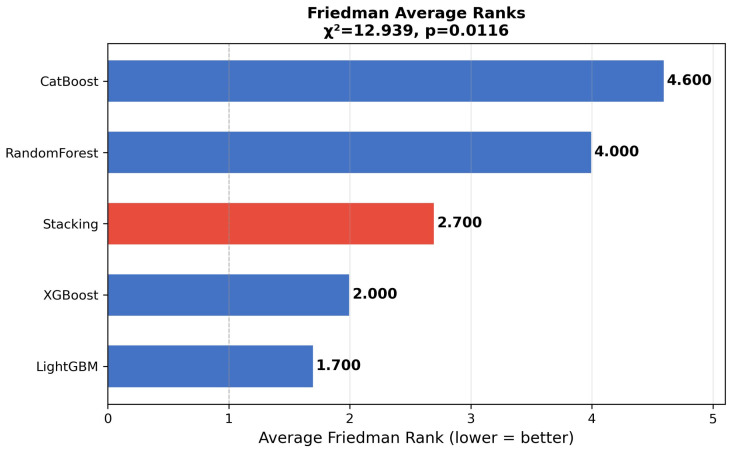
Average Friedman ranks across classifiers (χ^2^ = 12.939, *p* = 0.0116). The red bar indicates the proposed stacking ensemble.

As shown in Table 18, the Friedman test resulted in a statistically significant outcome (χ^2^ = 12.939, *p* = 0.0116). Hence, the null hypothesis was rejected, implying that all classifiers did not perform equally. Mean ranks based on the Friedman test are provided in Table 19 and are graphically visualized in Figure 24. LightGBM yielded the highest mean rank (1.700), followed by XGBoost (2.000) and the proposed stacking model (2.700), which occupied the third place among all folds. Random Forest and CatBoost yielded the lowest mean ranks equal to 3.100 and 4.000, respectively.

**Figure 25:**
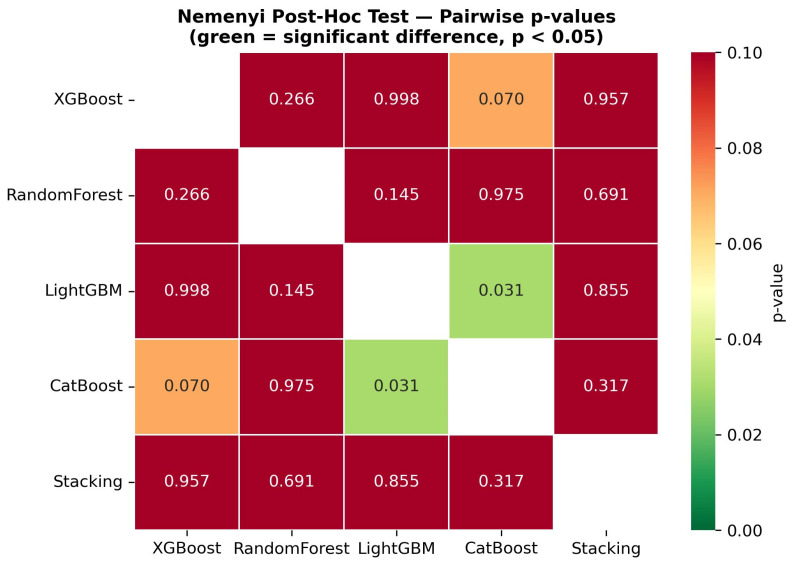
Nemenyi post hoc test heatmap. Green cells indicate statistically significant pairwise differences (*p* < 0.05).

Nemenyi post hoc analysis (in Figure 25) revealed that LightGBM performed significantly better than CatBoost (*p* = 0.031; Table 20). None of the remaining pairwise comparisons reached statistical significance. Such an outcome was anticipated because of insufficient statistical power caused by a relatively small number of folds (*n* = 5). Therefore, the lack of significant differences between stacking and its components should not be interpreted as similarity. Instead, it implies that larger samples are necessary to obtain statistically significant results. To address this issue, a pairwise Wilcoxon signed-rank test and a paired *t*-test were performed between the stacking ensemble and each component model, with effect size being quantified using Cohen’s d (Table 21). No pairwise comparison reached statistical significance due to low statistical power in *n* = 5 folds. Nevertheless, Cohen’s d indicated medium to large practical effect sizes of the difference between Stacking and CatBoost (d = 0.766) and Stacking and Random Forest (d = 0.642). Such effect sizes imply substantial differences between the ensemble and its components. Stacking and XGBoost, as well as Stacking and LightGBM, produced small negative effect sizes (d = −0.356 and d = −0.467, respectively).

## 5. Discussion

An explainable ML-based classification framework was proposed for the assessment of CTS severity based on clinical and demographic features. A highly efficient classification pipeline with the use of the ADASYN oversampling strategy, combined with advanced feature engineering techniques and a stacking approach, was developed. While the use of MixUp resulted in exceptionally high accuracy, it was concluded that these results did not correspond to real-life generalization due to overfitting. Over-saturation made it impossible for MixUp to create synthetic observations that do not belong to the test set; consequently, it created very similar data points to those included in the test set. Moreover, KDE, t-SNE, and PCA results indicated that despite preserved correlations, the marginal distributions were distorted. Thus, the accuracy metric in the case of MixUp is likely to be affected by overfitting. Contrary to MixUp, ADASYN proved its usefulness in achieving high accuracy (0.9045), maintaining marginal distributions and correlation structure, and retaining SHAP order consistent with the expected severity ranking (CSA, thenar weakness, symptom duration). As a result, ADASYN was used to augment the samples.

In addition to competitive overall accuracy, the use of LightGBM stacking helped to maintain balanced performance metrics across all severity classes. Although the stacking ensemble did not consistently improve mean accuracy over the best individual base model (stacking: 91.15% vs. XGBoost: 90.49% on the test set), it demonstrated superior calibration for the clinically critical Severe class (ECE = 0.024) and reduced prediction variance across folds (SD = 0.031 vs. SD = 0.027 for the best single model), indicating more stable and reliable predictions across different patient subsets. As noted above, initially, there was a problem with the unbalanced dataset; thus, a substantial boost in performance metrics was achieved through ADASYN augmentation. Moreover, the ability of ADASYN to improve recall and F1-score across severity classes proves its utility in overcoming class imbalance in clinical datasets. In the context of model deployment, the interpretability of the final results was deemed essential. Both SHAP and LIME analysis revealed clinically interpretable parameters: CSA, symptom duration, pain score (NRS), and palmar bowing (PB). Concerning the use of stacking, the positive effect of probability fusion via the LightGBM meta-learner was observed in terms of calibration quality and cross-class consistency. It should be mentioned that probability-based meta-features dominated the SHAP and permutation importance scores, which indicated the usefulness of ensemble techniques in capturing complementary decision boundaries across base models.

**External Validation and Generalizability:** It is worth mentioning a potential limitation of the proposed work in the context of generalizability. First, publicly available dataset [6] was used for both training and testing. Prior to augmentation, 305 samples were held out as the test set; ADASYN was applied exclusively to the remaining 732 training samples, yielding 1216 balanced training instances—corresponding to an 80/20 train/test ratio (1216:305) relative to the final augmented dataset (*n* = 1521). Though this held-out test set provided an unbiased estimate of model performance within the studied population, these results cannot be guaranteed to generalize to new datasets or different clinical settings. Therefore, any performance metrics should be treated as characterizing the behavior of the developed models on the studied cohort, but not others.

There are multiple sources of inter-center variance in the use of CTS-related clinical variables that make generalization difficult. In particular, CSA values strongly depend on ultrasound machine parameters (probe frequency and brand), sonographer’s expertise (measurement location, patient wrist position, and probe pressure), and clinical protocol for median nerve boundary determination. Another ultrasound-related feature, palmar bowing (PB), is a reproducible measurement, yet it may vary with changes in equipment settings and the sonographer’s skill. NRS pain rating is prone to inter-individual variability and subjectivity, which might be affected by factors such as the culture and linguistic background of patients. Finally, the distribution of the studied group’s age, sex, comorbidity (especially diabetes), and average symptom duration might not represent all other CTS populations. If applied to different centers, a model trained on this distribution might suffer from a loss of performance.

To address the mentioned limitations, the main idea for the future is to conduct an external prospective multi-center validation of the proposed models. To make the validation easier in the future, a limited set of 11 variables (those routinely collected in CTS assessment) was chosen for model construction and development. Further validation efforts should include checking the consistency of model calibration and class-level performance metrics (especially of Class 1), along with the collection of equipment- and sonographer-level covariates as possible sources of variation.

**Multiparametric Ultrasound Assessment of CTS:** An emphasis is placed on CSA among other variables in the presented study; this is due to the fact that CSA remains the most validated and standardized parameter used in CTS ultrasound assessment. Nonetheless, multiparametric ultrasound assessment must be considered in the context of modern CTS assessment guidelines. Apart from CSA, several ultrasound variables are currently included in the assessment of CTS severity. Nerve echogenicity—defined as the decreased intraneural echogenicity as a sign of nerve edema—is related to CTS severity independently of nerve enlargement. Intraneural vascularity measured using Doppler imaging is associated with CTS severity in terms of active nerve inflammation. Nerve excursion/mobility can serve as a measure of functional severity, as nerve restriction indicates fibrosis. Flattening ratio and palmar bowing of the flexor retinaculum—which was included in the current dataset—are geometric indicators of the entrapment severity. Taken together, all these parameters should provide a more detailed insight into the nature of median nerve entrapment.

The major drawback of the presented model lies in the lack of some ultrasound variables in the source dataset. The authors did not have the opportunity to incorporate echogenicity, vascularity, Doppler score, and nerve longitudinal excursion as model input features. Such an inclusion is expected to enhance the model’s discriminatory power and help with the differentiation of moderate CTS cases from severe ones (where the overlap in CSA values was observed). A full multiparametric ultrasound CTS dataset would allow for the creation of more robust machine learning models for CTS severity assessment.

**Clinical Decision Mapping and Practical Utility:** An essential aspect in the use of machine learning in any medical problem is the possibility of translating model outputs into action. In the case under discussion, the three classes defined by the developed models directly correlate with the existing management strategies in CTS management. In mild CTS (Class 0), conservative therapy should be initiated: nighttime splints for the wrist, changes in activities, physiotherapy, and non-steroid medications; EMG/NCS is optional at this stage. A model may prove its efficiency by confirming the conservative treatment plan and preventing excessive specialist referrals. In moderate CTS (Class 1), corticosteroid injections are required; EMG/NCS should confirm electrophysiological severity. The model can be useful for directing patients towards injections and scheduling EMG/NCS. In severe CTS (Class 2), surgical treatment—open or endoscopic release—is necessary. EMG/NCS should also be done pre-operatively. At this stage, the model helps to initiate referral to surgery, which is especially useful in healthcare contexts with long specialist referral waits.

Regarding the relationship between the machine learning model and EMG/NCS assessments, the proposed model is not supposed to replace the former. Nevertheless, it may be used as (a) a preliminary screening tool in primary care facilities or resource-limited settings where specialist referrals may not be possible; (b) a means of standardization that will eliminate sonographer-to-sonographer variations in severity grading using ultrasound; (c) an assistance tool at point-of-care that helps to decide about further electrophysiological assessment. It is important that clinical error analysis supports the proposed positioning of the models. Indeed, according to the confusion matrix, only 1.0% of cases were classified critically incorrectly (three severe patients predicted as mild cases); no mild CTS patients were predicted as severe (and, therefore, referred for surgery erroneously). The remaining 9.5% of predictions are classified as adjacent-class errors and correspond to a minor change in treatment plan. Mild and moderate classes have some overlap as they share treatment options.

The clinical significance of the constructed models was additionally supported by the high statistical significance of most of the considered features in all severity groups. Among those, the most significant are CSA, BMI, PB, and symptom duration, which correspond to the knowledge from the current literature. One may note a strong agreement with [6,17]. In particular, they demonstrate that sonographic and clinical parameter combination results in much more reliable CTS severity classification. In terms of methodology, this study is a part of the emerging field of machine learning applications in musculoskeletal medicine.

## 6. Conclusions

In this work, we have designed an interpretable AI-based framework that predicts CTS severity using the clinical and demographic derived variables. For overcoming the problem of data imbalance, the ADASYN approach has been applied. Furthermore, we constructed the stacking ensemble, consisting of four base learners such as XGBoost, Random Forest, LightGBM, and CatBoost. As a result, the obtained model shows satisfactory accuracy of prediction and comparable performance on each severity class. By applying the calibration procedure, the stacked ensemble achieved 91.15% accuracy and the macro-F1 score of 91.13% in the out-of-sample test set for the presented cohort of patients. It is worth mentioning that these results are obtained for a single dataset analysis, and thus, independent multi-center validation of the model is needed in order to determine its generalizability potential.

Explaining model predictions by using SHAP and LIME methods, it was found that the model works by taking into account the clinically accepted predictors, with the following features having the highest influence on the model output: cross-sectional area (CSA), the duration of symptoms, NRS pain score, and palmar bowing. In other words, the proposed framework is based on the features that are widely discussed in the clinical literature. Moreover, it should be mentioned that severity classes suggested by our framework correspond to the current guidelines on CTS severity levels that imply certain treatment options, i.e., conservative treatment, corticosteroid injection and surgical intervention. Thus, the potential application of the model lies in ultrasound-based assessment and decision-making in the context of CTS diagnosis in ultrasound facilities. As the model showed a low critical error rate (1.0%), no unnecessary surgeries were referred. Furthermore, the model does not have the purpose to substitute the EMG/NCS tests, but to become a complementary tool for them. The following directions of future research are recommended. First, it will be necessary to conduct the multi-center external validation study of the model. Second, the future datasets will contain information about the whole spectrum of multiparametric ultrasound features, such as the nerve echogenicity, the presence/absence of the nerve vascularity (Doppler imaging) and mobility of the nerve along its longitudinal axis, apart from CSA and PB measurements. Third, it is expected that electrophysiological and biomechanical features will be added to the dataset in order to improve the classification accuracy of the model, especially of the moderate class.

## Figures and Tables

**Table 1:** Summary of prior studies on CTS diagnosis and classification performance.

Ref.	Method	Dataset	Evaluation Metrics
[6]	Machine Learning, Random Up-Sampling and SMOTE 10-fold cross-validation,	The data set contains 1037 CTS data containing 11 features. Of these, 507 represent the hand with mild, 276 moderate and 254 severe CTS.	XGB overall accuracy 76.6 (71.2–81.5)% max
[20]	DL	50 healthy (22 males and 28 females) and 50 patients (19 males and 31 females).	Specificity of 1.00
[21]	CNN and SVM 10-fold cross-validation	100 CTS and 100 normal wrist images.	CNN 0.980 AUC, SVM 0.943 AUC
[22]	DeepNerve method ConvLSTM + U-Net + MaskTrack 4-fold cross-validation	Twenty-four image sequences were captured from six male participants. Each image sequence consists of approximately 420 frames.	F-score 0.9015
[23]	DeepLabV3+, U-Net, FPN and Mask-R-CNN	The dataset included 52 participants, of whom 36 were used for training and 16 for testing based on their varying visual characteristics.	DeepLabV3+ and Mask R-CNN IoU 0.83
[24]	MNT-DeepSL	Ultrasound images were collected from 100 hands (left and right) of 50 individuals, with six wrist movements recorded for each hand.	MNT-DeepSL Accuracy 0.9
[25]	Machine Learning (autoencoder (AE))	There were 36 participants, 36 hands with CTS and 27 hands without CTS.	AUC of 0.86
[26]	LR, SVM, kNN, DT, NB Feature extraction, K-fold cross-validation (leave-one-out cross-validation).	Data generated by the signals of 65 hands. The set (46 CTS and 19 control) comprised 302 features.	The SVM model achieved 95% neurophysiological and 89% clinical diagnostic accuracy
[27]	KNN, DVM, XGB, YSA 75/25 segmentation, SMOTE Grid Search Optimization	Data were obtained from a registry comprising records of 1919 consecutive patients who underwent CTD (Carpal Tunnel Decompression) for CTS (Carpal Tunnel Syndrome).	XGB/Accuracy 0.759
[28]	U-NET and Deep CTS, Image Processing, 5-fold cross-validation	The dataset consists of 415 pairs of images and labels, including both left and right hands.	0.63 accuracy of intersection over union
**Our study**	**Machine Learning** **ADASYN data augmentation** **5-fold cross-validation**	**1521 data with 3 classes**	**Final stacking model accuracy 0.9115**

**Table 2:** A summary table of baseline demographic and clinical features of the data.

Variable	Mild (*n* = 507)	Moderate (*n* = 276)	Severe (*n* = 254)	*p*-Value
Age (years, mean ± SD)	57.3 ± 10.6	59.2 ± 10.8	57.8 ± 11.2	0.069
Male (%)	39.2	44.6	32.7	0.183
BMI (kg/m^2^)	24.2 ± 3.4	24.7 ± 3.0	25.8 ± 3.7	<0.001
Diabetes prevalence (%)	9.3	16.3	21.6	<0.001
Symptom duration (months)	4.3 ± 5.0	8.5 ± 8.2	15.9 ± 12.8	<0.001
NRS pain score	3.3 ± 1.3	4.9 ± 1.5	6.1 ± 1.5	<0.001
Thenar weakness/atrophy (%)	0.2	8.7	66.5	<0.001

**Table 3:** Data preprocessing flow: Sample counts and feature dimensionality at each stage.

Stage	Total N	Mild	Moderate	Severe	Features	Note
1. Raw Dataset [6]	1037	507	276	254	11	Public dataset
2. After Preprocessing	1037	507	276	254	11	No missing values
3. Train/Test Split (80/20)	732	358	195	179	11	Test set: 305 samples (held out)
4. After ADASYN (train only)	1216	406	405	405	11	Synthetic minority oversampling
5. After Feature Engineering	1216	406	405	405	55	Polynomial + interaction + binning

**Table 4:** The descriptive statistics of the features.

Index	Count	Mean	Std	Min	%25	%50	%75	Max	Skew	Kurtosis	N_Missing	Pct_Missing
**Age**	1216	58.0559	10.5921	19.0	52.0	58.0	64.0	87.0	−0.0991	0.2468	0	0.0
**BMI**	1216	24.8325	3.1938	17.7095	22.6222	24.4076	26.7782	42.5980	0.7086	1.1311	0	0.0
**CSA**	1216	15.5201	4.2296	7.0	13.0	15.0	17.6907	37.0	1.2229	2.7762	0	0.0
**PB**	1216	2.5877	1.7483	0.5	1.8	2.3767	3.0080	30.0	8.9990	111.2309	0	0.0
**Duration**	1216	8.8133	9.2346	0.0	3.0	5.0	12.0	60.0	1.8296	3.4730	0	0.0
**NRS**	1216	4.5953	1.7032	1.0	3.0	5.0	6.0	10.0	0.1787	−0.1683	0	0.0
**Sex**	1216	0.5189	0.4998	0.0	0.0	1.0	1.0	1.0	−0.0758	−1.9975	0	0.0
**Side**	1216	0.3700	0.4830	0.0	0.0	0.0	1.0	1.0	0.5388	−1.7124	0	0.0
**Diabetes**	1216	0.0970	0.2961	0.0	0.0	0.0	0.0	1.0	2.7259	5.4398	0	0.0
**NP**	1216	0.4580	0.4984	0.0	0.0	0.0	1.0	1.0	0.1685	−1.9748	0	0.0
**Weakness**	1216	0.1973	0.3981	0.0	0.0	0.0	0.0	1.0	1.5225	0.3188	0	0.0
**Severity**	1216	0.9991	0.8170	0.0	0.0	1.0	2.0	2.0			0	0.0

**Table 5:** Statistical tests of feature associations with target variable (Severity).

Feature Type	Feature	Test Applied	*p*-Value	Significance (*α* = 0.05)
**Numerical**	Age	Kruskal–Wallis	0.3165	Not Significant
	BMI	Kruskal–Wallis	<0.0001	Significant
	CSA	Kruskal–Wallis	<0.0001	Significant
	PB	Kruskal–Wallis	<0.0001	Significant
	Duration	Kruskal–Wallis	<0.0001	Significant
	NRS	Kruskal–Wallis	<0.0001	Significant
**Categorical**	Sex	Chi-square	0.0011	Significant
	Side	Chi-square	0.0001	Significant
	Diabetes	Chi-square	0.0007	Significant
	NP	Chi-square	<0.0001	Significant
	Weakness	Chi-square	<0.0001	Significant

**Table 6:** Ten-classifier comparison of ADASYN-balanced dataset (5-fold CV). ★ = Selected base models.

Model	Accuracy (Mean ± SD)	F1-Macro (Mean ± SD)	Status
LightGBM ★	0.8890 ± 0.0329	0.8894 ± 0.0323	SELECTED—top accuracy
XGBoost ★	0.8808 ± 0.0284	0.8812 ± 0.0280	SELECTED—consistent performance
Random Forest ★	0.8799 ± 0.0433	0.8810 ± 0.0420	SELECTED—diversity for ensemble
MLP	0.8652 ± 0.0237	0.8649 ± 0.0238	Not selected—lower accuracy
CatBoost ★	0.8561 ± 0.0369	0.8571 ± 0.0359	SELECTED—categorical handling advantage
Decision Tree	0.7541 ± 0.0320	0.7568 ± 0.0292	Not selected
SVM (RBF)	0.7385 ± 0.0331	0.7404 ± 0.0334	Not selected
Logistic Regression	0.6957 ± 0.0436	0.6966 ± 0.0427	Not selected
K-Nearest Neighbors	0.6891 ± 0.0421	0.6903 ± 0.0421	Not selected
Naive Bayes	0.5164 ± 0.0194	0.4803 ± 0.0271	Not selected

**Table 7:** Hyperparameter summary for base models and final stacking meta-learner.

Hyperparameter Summary for Models		
Models	Parameters	Values
**XGBoost**	learning_rate	0.0496
	n_estimators	450
	max_depth	8
	subsample	0.765
	colsample_bytree	0.543
**Random Forest**	n_estimators	550
	max_depth	26
	max_features	‘log2’
**LightGBM (base model)**	learning_rate	0.0798
	n_estimators	500
	num_leaves	40
	max_depth	6
	subsample	0.6005
**CatBoost (base model)**	learning_rate	0.0707
	iterations	450
	depth	7
**Meta-Learner (Stacking LGBM)**	learning_rate	0.00394
	n_estimators	600
	max_depth	9
	num_leaves	70
	subsample	0.782

**Table 8:** Performance summary of individual models and final stacking ensemble on test set.

Model	Accuracy	Precision (Macro)	Recall (Macro)	F1-Score (Macro)	Balanced Accuracy	ROC AUC (Macro)	MCC	Cohen’s Kappa
**XGBoost**	0.9049	0.9051	0.9049	0.9048	0.9049	0.9695	0.8576	0.8574
**Random Forest**	0.9016	0.9018	0.9017	0.9009	0.9017	0.9731	0.8533	0.8525
**LightGBM**	0.9049	0.9057	0.9049	0.9051	0.9049	0.9671	0.8576	0.8574
**CatBoost**	0.9049	0.9044	0.9049	0.9046	0.9049	0.9774	0.8575	0.8574
**Final Stacking**	**0.9115**	**0.9112**	**0.9115**	**0.9113**	**0.9115**	**0.9708**	**0.8672**	**0.8672**

**Table 9:** Per-class precision (P), recall (R), and F1-score for each model used on the test set (*n* = 305).

Model	Mild P	Mild R	Mild F1	Mod P	Mod R	Mod F1	Sev P	Sev R	Sev F1
XGBoost	0.877	0.921	0.899	0.879	0.853	0.866	0.960	0.941	0.951
Random Forest	0.857	0.891	0.874	0.822	0.814	0.818	0.960	0.931	0.945
LightGBM	0.877	0.921	0.899	0.880	0.863	0.871	0.970	0.941	0.955
CatBoost	0.811	0.891	0.849	0.846	0.755	0.798	0.952	0.961	0.956
Stacking (Proposed)	0.839	0.931	0.883	0.884	0.824	0.853	0.969	0.931	0.950

**Table 10:** Meta-learner comparison on OOF meta-features (5-fold CV).

Meta-Learner	Accuracy (Mean ± SD)	F1-Macro (Mean ± SD)	Selection Rationale
Logistic Regression	0.8849 ± 0.0203	0.8855 ± 0.0196	Linear—may underfit ensemble interactions
Random Forest	0.8939 ± 0.0202	0.8944 ± 0.0194	Highest mean accuracy; higher variance
XGBoost	0.8882 ± 0.0039	0.8883 ± 0.0036	Very low variance but moderate accuracy
LightGBM (Selected)	0.8865 ± 0.0139	0.8864 ± 0.0137	Optimal accuracy–stability balance; selected

**Table 11:** Summary of dataset and final model performance.

Category	Metric/Value
**Total Samples**	1521
**Features (Final)**	55
**Classes**	3
**Training Samples**	1216
**Test Samples**	305
**Final Model Accuracy**	91.15%
**Final Model F1-Score**	91.13%
**Final Model ROC AUC**	0.9708
**Best Individual Model**	XGBoost (90.49% Acc)
**Misclassified Samples**	27

**Table 12:** SHAP–LIME consistency results based on Spearman rank correlation (*n* = 50 test instances). * = Statistically significant (*p* < 0.05).

Metric	Value	*p* -Value	Top-15 Overlap	N Instances
Spearman ρ (SHAP vs. LIME global ranks)	0.2808	0.036 *	5/15	50

**Table 13:** Clinical impact summary for the stacking ensemble on the test set (*n* = 305). Critical errors—defined as Severe ↔ Mild misclassifications—constitute only 1.0% of all predictions. No Mild patients were misclassified as Severe, indicating zero unnecessary surgical referrals.

Error Type	Count	Rate (%)	Clinical Implication
Correct classification	273	89.5%	No clinical impact
Moderate impact (adjacent class)	29	9.5%	Minor treatment adjustment needed
Critical error (Severe ↔ Mild)	3	1.0%	Surgical patient missed or overtreated
Severe predicted as Mild (worst case)	3	1.0%	Surgery delayed—highest clinical cost
Mild predicted as Severe	0	0.0%	Unnecessary surgery referral—none occurred

**Table 14:** Ablation study results (mean ± SD, 5-fold CV). Feature engineering (C1 → C3) is the dominant contributor, yielding a +13.2% accuracy improvement. Stacking (C3 → C4) provides prediction stability and balanced per-class performance.

Configuration	Accuracy	F1-Macro	MCC	ROC-AUC
C1: Baseline (No ADASYN, No FE, No Stacking)	0.756 ± 0.030	0.730 ± 0.033	0.600 ± 0.043	0.890 ± 0.014
C2: +ADASYN (No FE, No Stacking)	0.755 ± 0.020	0.733 ± 0.022	0.610 ± 0.033	0.884 ± 0.016
C3: +Feature Engineering (No Stacking)	0.887 ± 0.027	0.888 ± 0.026	0.835 ± 0.038	0.973 ± 0.007
C4: +Stacking (Proposed—Full Pipeline)	0.884 ± 0.031	0.885 ± 0.030	0.827 ± 0.045	0.945 ± 0.013

**Table 15:** Augmentation strategy comparison (5-fold CV, within-fold application, and original imbalanced dataset, *n* = 1037). ADASYN achieves the highest accuracy, F1-Macro, Severe Recall, and MCC across all evaluated strategies.

Strategy	Accuracy	F1-Macro	Severe Recall	MCC
No Augmentation	0.748 ± 0.023	0.722 ± 0.026	0.776 ± 0.026	0.596 ± 0.039
Class-Weight (Balanced)	0.747 ± 0.035	0.727 ± 0.036	0.791 ± 0.044	0.598 ± 0.058
SMOTE	0.747 ± 0.023	0.725 ± 0.026	0.795 ± 0.015	0.597 ± 0.039
ADASYN (Proposed)	0.755 ± 0.022	0.734 ± 0.024	0.811 ± 0.035	0.611 ± 0.036

**Table 16:** Training vs. cross-validation accuracy gap per model. The observed gap (~0.11) is consistent with the known artifact of ADASYN synthetic oversampling and does not indicate true overfitting.

Model	Train Acc (Mean ± SD)	CV Acc (Mean ± SD)	Gap
XGBoost	1.0000 ± 0.0000	0.8873 ± 0.0266	0.1127
RandomForest	0.9883 ± 0.0026	0.8684 ± 0.0468	0.1199
LightGBM	1.0000 ± 0.0000	0.8923 ± 0.0290	0.1077
CatBoost	0.9852 ± 0.0026	0.8676 ± 0.0306	0.1176
Stacking (meta)	0.9992 ± 0.0004	0.8923 ± 0.0163	0.1069

**Table 17:** Five-fold cross-validation accuracy scores per model. Mean and SD are computed across folds.

Model	Fold 1	Fold 2	Fold 3	Fold 4	Fold 5	Mean	SD
LightGBM	0.8770	0.8436	0.9095	0.9053	0.9259	0.8923	0.0290
XGBoost	0.8893	0.8436	0.9012	0.8930	0.9218	0.8873	0.0257
Stacking (Proposed)	0.8893	0.8313	0.8724	0.9136	0.9136	0.8840	0.0306
RandomForest	0.8730	0.7778	0.8930	0.8889	0.9095	0.8684	0.0468
CatBoost	0.8648	0.8148	0.8889	0.8642	0.9053	0.8676	0.0306

**Table 18:** Friedman test results. The significant outcome (*p* = 0.0116) justifies post hoc pairwise analysis.

Test	χ^2^ Statistic	*p* -Value	Decision
Friedman Test (k = 5 classifiers, *n* = 5 folds)	12.939	0.0116	Reject H_0_ (*p* < 0.05)

**Table 19:** Average Friedman ranks per model (lower = better). The stacking ensemble ranks third overall, remaining consistently competitive across all five folds.

Model	Average Rank	Interpretation
LightGBM	1.700	Best-ranked classifier
XGBoost	2.000	Second best
Stacking (Proposed)	2.700	Third—consistently competitive
RandomForest	4.000	Fourth
CatBoost	4.600	Fifth (NaN-affected folds imputed)

**Table 20:** Nemenyi post hoc pairwise *p*-values. * = Statistically significant difference (*p* < 0.05). LightGBM significantly outperforms CatBoost (*p* = 0.031); no other pairs reach significance, consistent with the limited statistical power of five-fold evaluation.

Model	XGBoost	RandomForest	LightGBM	CatBoost	Stacking
XGBoost	—	0.266	0.998	0.070	0.957
RandomForest	0.266	—	0.145	0.975	0.691
LightGBM	0.998	0.145	—	0.031 *	0.855
CatBoost	0.070	0.975	0.031 *	—	0.317
Stacking	0.957	0.691	0.855	0.317	—

**Table 21:** Pairwise statistical comparisons between the stacking ensemble and each base model. Cohen’s d values indicate medium-to-large practical effect sizes for Stacking vs. CatBoost (d = 0.766) and Stacking vs. Random Forest (d = 0.642), suggesting practical relevance despite non-significance.

Comparison	ΔAcc	Wilcoxon W	*p* (Wilc.)	t-Stat	*p* (*t*-Test)	Cohen’s d
Stacking vs. XGBoost	−0.0058	3.0	0.465	−0.713	0.516	−0.356
Stacking vs. RandomForest	+0.0156	3.0	0.313	1.284	0.268	0.642
Stacking vs. LightGBM	−0.0082	3.0	0.313	−0.934	0.403	−0.467
Stacking vs. CatBoost	+0.0164	2.0	0.188	1.533	0.200	0.766

## Data Availability

Data are publicly available as given in the dataset section and are accessible via https://www.nature.com/articles/s41598-021-97043-7 (accessed on 3 February 2025).
